# Supplementary material for: Active species in chloroaluminate ionic liquids catalyzing low-temperature polyolefin deconstruction
Source: Nat Commun. 2024 Jul 10;15:5785. doi: 10.1038/s41467-024-49827-4 (PMC11237162; doi:10.1038/s41467-024-49827-4)
Supplement: Supplementary file 1 — Supplementary Information [file 41467_2024_49827_MOESM1_ESM.pdf]

Supplementary Information

Active species in chloroaluminate ionic liquids catalyzing low-temperature polyolefin deconstruction

Wei Zhang,[1,2]* Rachit Khare,[2] Sungmin Kim,[1 2] Lillian Hale,[1] Wenda Hu,[1] Chunlin Yuan,[2] Yaoci Sheng,[2] Peiran Zhang,[2] Lennart Wahl,[2] Jiande Mai,[1] Boda Yang,[1] Oliver Y. Gutiérrez,[1] Debmalya Ray,[1] John Fulton,[1] Donald M. Camaioni,[1] Jianzhi Hu,[1,3] Huamin Wang,[1] Mal-Soon Lee[1] and Johannes A. Lercher[1,2]*

[1] Institute for Integrated Catalysis, Pacific Northwest National Laboratory, P.O. Box 999, Richland, WA99352, USA
[2] Department of Chemistry and Catalysis Research Center, Technische Universität München, Lichtenbergstr. 4, 85747, Garching, Germany
[3] The Gene and Linda Voiland School of Chemical Engineering and Bioengineering, Washington State University, Pullman, WA 99164, USA

*To whom correspondence should be addressed: Wei Zhang (wzhangx@outlook.com); Johannes A. Lercher (johannes.lercher@ch.tum.de)

Table of Contents

Supplementary Fig. 1 | Relationship between initial LDPE conversion rate, the mole fraction (χ) of $Al_2Cl_7^-$, and empirical [$AlCl_3$] concentration (K*[$AlCl_3$]) 4

Supplementary Note 1: The X-ray absorption spectroscopy and evaluation of chloroaluminate species in ionic liquids....................5

Supplementary Fig. 2 | The X-ray absorption spectroscopy and quantitative evaluation of chloroaluminate species in ionic liquids. 6

Supplementary Table 1. Fitting parameters for the EXAFS of [C_4Py]Cl-$x$$AlCl_3$ ionic liquids with varying $AlCl_3$:[C_4Py]Cl molar ratios....................7

Supplementary Fig. 3 | The corresponding Al–Cl coordination numbers and interatomic distances obtained from EXAFS fit....................7

Supplementary Fig. 4 | Al K-edge EXAFS of [C4Py]Cl-xAlCl$_3$ ionic liquids (x=1, 1.3, and 1.4). 8

Supplementary Fig. 5 | Al K-edge EXAFS of [C_4Py]Cl-$x$$AlCl_3$ ionic liquids (x=1.5, 1.6, and 1.7). 9

Supplementary Fig. 6 | Al K-edge EXAFS of [C_4Py]Cl-$x$$AlCl_3$ ionic liquids (x=1.8, 1.9, and 2).10

Supplementary Fig. 7 | Temperature-programmed ^{27}Al MAS NMR spectroscopy on chloroaluminate ionic liquids....................11

Supplementary Fig. 8 | DFT-NMR modeling of $Al_2Cl_7^-$ showing a broadened linewidth in the ^{27}Al MAS NMR spectra 12

Supplementary Fig. 9 | Raman spectra of [C_4Py]Cl-$x$$AlCl_3$ (x=1-2)....................13

Supplementary Note 2: Derivations of concentration of $AlCl_4^-$ and $Al_2Cl_7^-$ ($Al_2Cl_7^- \rightarrow AlCl_4^- + AlCl_3$) over various time intervals (t) 14

Supplementary Fig. 10 | The computational simulation of the dissociation of $Al_2Cl_7^-$ in a DCM solvent. 17

Supplementary Fig. 11 | Chloroaluminate transformation in the presence of TBC 18

Supplementary Fig. 12 | Raman spectroscopy analysis of the interaction between TBC and [C_4Py]Cl-xAlCl$_3$.. 19

Supplementary Fig. 13 | The conversion of LDPE plotted against the corresponding variation of chloroaluminate species in ionic liquids 20

Supplementary Fig. 14 | In situ Raman spectra recorded during the cracking-alkylation of n-$C_{16}H_{34}$ and iC$_5$ in the presence of TBC....................21

Supplementary Fig. 15 | The computational simulations of $Al_2Cl_7^-$ catalyzed hydride abstraction from iC$_5$. 22

Supplementary Fig. 16 | Computational simulation of $AlCl_3$-TBC adduct catalyzing hydride abstraction from nC_{16}. 23

Supplementary Fig. 17 | In situ 1H NMR spectra of tandem cracking-alkylation of n-C_{16} and iC_5 over $[C_4Py]Cl$-$2AlCl_3$..24

Supplementary Fig. 18 | Raman spectroscopy analysis of the interaction between TBC and $[C_4Py]Cl$-$2AlCl_3$. 25

Supplementary Fig. 19 | Time-resolved conversion profile of LDPE in the presence of TBC or $C_5^=$ as additives, compared to conditions without additives...26

Supplementary Fig. 20 | Computed reaction pathway for the formation of $AlCl_3$-$C_5^=$ adduct. 27

Supplementary Fig. 21 | Operando IR setup combing a ReactIRTM 45m spectrometer.........28

Supplementary Fig. 22 | Operando IR spectra were recorded during LDPE depolymerization in the absence of iC_5 over $[C_4Py]Cl$-$2AlCl_3$..29

Supplementary Fig. 23 | Three-dimensional operando IR spectra of the *sp*^{3} C–H vibrations as a function of reaction time, recorded during LDPE depolymerization in the presence of iC_5 over $[C_4Py]Cl$-$2AlCl_3$ at 60 °C 30

Supplementary Fig. 25 | Operando IR spectra of the iC_5 on-off experiment. 32

Supplementary Note 3: Derivations of rate equations in the cracking-alkylation of polyolefin with *i*C_5 over $[C_4Py]Cl$-$xAlCl_3$ 33

Supplementary Fig. 26 | Proposed sequence of key reaction steps in the cracking-alkylation of polyolefin with iC_5 over $[C_4Py]Cl$-$xAlCl_3$..33

Supplementary Fig. 27 | Kinetic analysis of cracking-alkylation cycles of polyolefin with iC_5.36

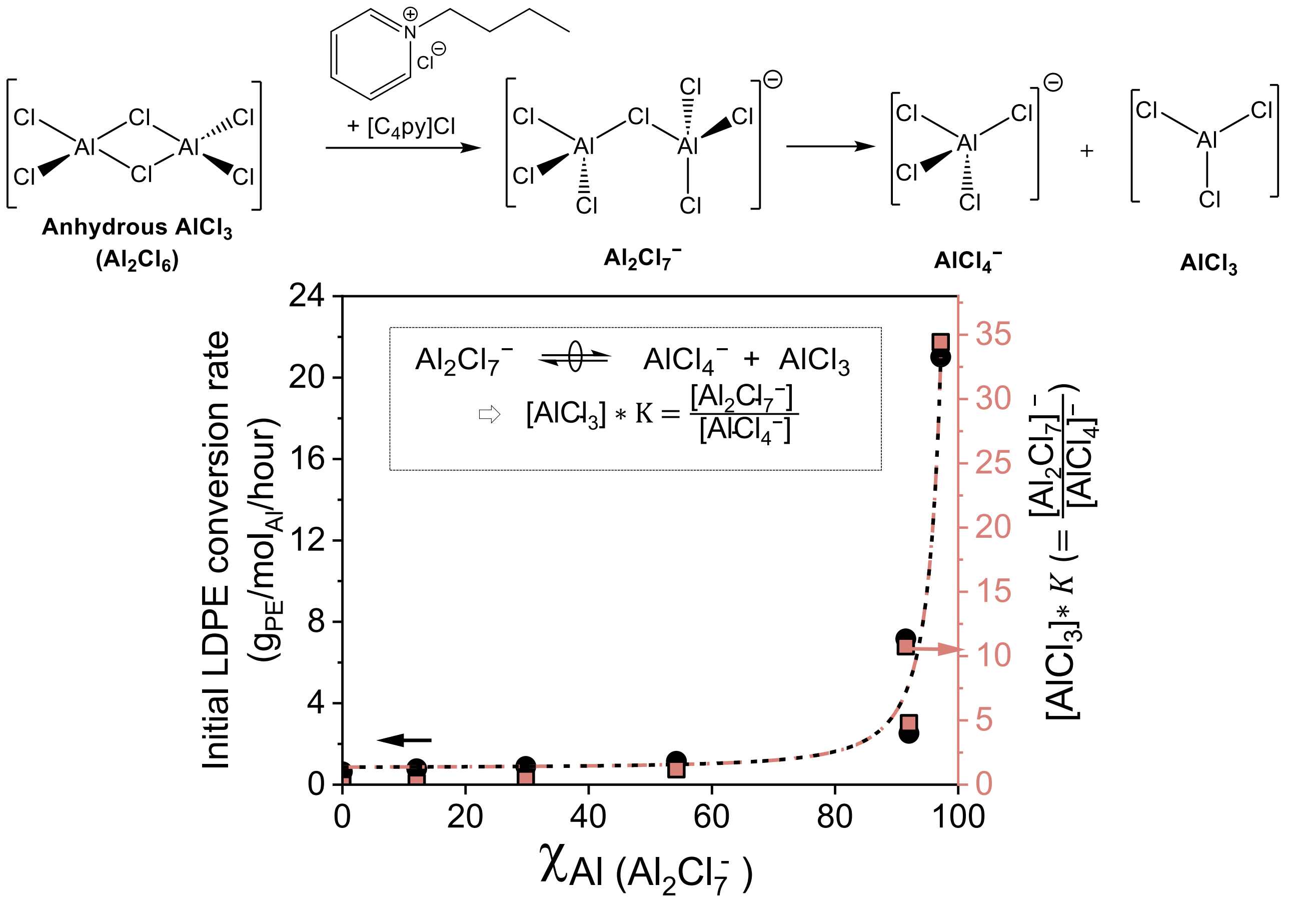

Supplementary Fig. 1 | Relationship between initial LDPE conversion rate, the mole fraction (χ) of $Al_2Cl_7^-$, and empirical [$AlCl_3$] concentration (K*[$AlCl_3$]).

Supplementary Note 1: The X-ray absorption spectroscopy and evaluation of chloroaluminate species in ionic liquids

Chloroaluminate ionic liquids were prepared by mixing anhydrous $AlCl_3$ with *N*-butyl pyridinium chloride at various ratios. The samples are denoted as ($[C_4Py]Cl$-$xAlCl_3$, wherein x represents the molar ratio of $AlCl_3$ and $[C_4Py]Cl$ (see the experimental section for details). Al K-edge X-ray absorption near edge structure (XANES, Supplementary Fig. 2a) of $[C_4Py]Cl$-$xAlCl_3$ ionic liquids showed a systematic increase in the pre-edge feature at ~1559.4 eV and a corresponding decrease in the intensity of the white line at ~1560.9 eV as the $AlCl_3/[C_4Py]Cl$ molar ratio increased. The increase in the intensity of the pre-edge feature can be attributed to a systematic change in the symmetry of Cl^- ions coordinating Al. The DFT geometry optimization (Supplementary Fig. 2b) suggests that the Al in the monomeric $AlCl_4^-$ species is symmetrically coordinated to four Cl^- ions with identical Al−Cl bond lengths (~2.16 Å). The Cl−Al−Cl bond angles (109.3°−109.7°) were close to the ideal tetrahedral bond angles (~109.5°). In the dimeric $Al_2Cl_7^-$, on the other hand, the coordination structure around Al is not symmetric. The Al−Cl bond length for six terminal Cl^- ions is ~2.12 Å, while the Al–Cl bond length for the bridging Cl^- is ~2.29 Å (denoted as Cl_{bridge}). In addition, the Cl−Al−Cl bond angles in $Al_2Cl_7^-$ vary between 102° – 114°, further showing the distorted tetrahedral symmetry in the dimer. The TDDFT-simulated X-ray absorption spectra of monomeric $AlCl_4^-$ and dimeric $Al_2Cl_7^-$ species are presented in Supplementary Fig. 1. The theoretical spectra confirm that the increase in the pre-edge feature and the corresponding decrease in white line can be attributed to the systematic formation of dimeric $Al_2Cl_7^-$ species within ionic liquids with higher $AlCl_3/[C_4Py]Cl$ molar ratio.

Supplementary Fig. 2c shows the k^2-weighted Fourier-transformed extended X-ray absorption fine structure (FT-EXAFS) of $[C_4Py]Cl$-$xAlCl_3$ ionic liquids with varying $[C_4Py]Cl:AlCl_3$ molar ratios. The FT-EXAFS shows a distinct feature at ~1.7 Å (phase-uncorrected) corresponding to Al–Cl scattering. A systematic broadening of this feature with increasing $[C_4Py]Cl:AlCl_3$ molar ratio suggests an increase in the Debye-Waller factor. A low Debye-Waller factor for ILs with $[C_4Py]Cl:AlCl_3$ = 1:1 is attributed to symmetric Al–Cl distances for all coordinated Cl atoms. On the other hand, in the dimeric $Al_2Cl_7^-$ species, the larger Al–Cl_{bridge} distance for the bridging Al atom

would contribute to an increase in the Debye-Waller factor and, thus, broadening of the Al–Cl scattering feature. This systematic increase in the Debye-Waller factor was also quantified by performing EXAFS fitting analysis (detailed fitting analysis is presented in Supplementary Figs. 3-6 and Supplementary Table 1). The fitting was performed using one Al−Cl single-scattering path obtained from the $AlCl_3$ crystal structure. The Al–Cl coordination number was fixed (= 4) during the fit. Supplementary Fig. 2d shows the fitted interatomic distances ($R_{Al–Cl}$) and Debye-Waller factors (σ^2) as a function of $[C_4Py]Cl:AlCl_3$ molar ratios. In agreement with the following NMR and Raman analysis in Fig. 2, the systematic increase in the Debye-Waller factor supports a systematic conversion of monomers to dimers as the $[C_4Py]Cl:AlCl_3$ ratio is increased from 1:1 to 1:2.

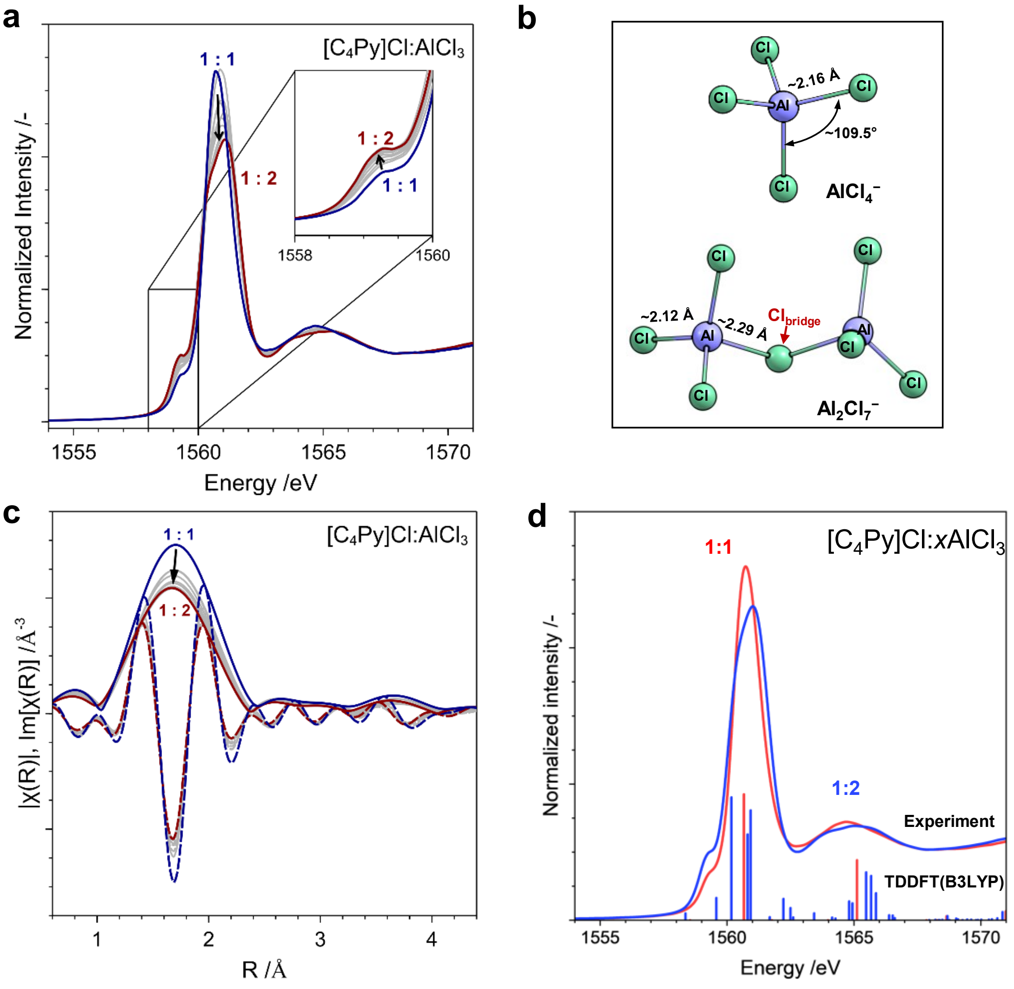

Supplementary Fig. 2 | The X-ray absorption spectroscopy and quantitative evaluation of chloroaluminate species in ionic liquids. **a**, Al K-edge XANES of $[C_4Py]Cl$-$x AlCl_3$ ionic liquids (x=1-2). **b,** B3LYP/def2-TZVP-optimized structures of $AlCl_4^-$ and $Al_2Cl_7^-$ ions. **c**, the k^2-weighted Fourier-transformed extended X-ray absorption fine structure (FT-EXAFS) of $[C_4Py]Cl$-$x AlCl_3$ ionic

liquids (x=1-2). **d**, B3LYP/def2-TZVP-simulated Al K-edge X-ray absorption spectra of $AlCl_4^-$ and $Al_2Cl_7^-$ anions. (Note: The experimentally obtained XANES of [C$_4$Py]Cl-xAlCl$_3$ ionic liquids with AlCl$_3$:[C$_4$Py]Cl equal to 1:1 and 1:2, respectively, are also shown for comparison. The simulated spectra were energy-shifted by 27.1 eV).

Supplementary Table 1. Fitting parameters for the EXAFS of [C$_4$Py]Cl-xAlCl$_3$ ionic liquids with varying AlCl$_3$:[C$_4$Py]Cl molar ratios. Fitting was performed in k-space simultaneously on the k^1-, k^2-, and k^3-weighted data in the k-range between k = 2.7 Å^{-1} and k = 8.0 Å^{-1}. The Al–Cl coordination number was fixed (= 4.0) during the fitting.

		Al–Cl			R-factor
**AlCl$_3$:[C$_4$Py]Cl**	**ΔE$_0$ /eV**	**CN[a]**	***d* /Å**	**$\sigma^2 \times 10^3$/Å^2**	
1:1.0	0.90 ± 3.21	4.0	2.126 ± 0.021	0.98 ± 1.26	0.043
1:1.3	0.85 ± 2.82	4.0	2.123 ± 0.020	3.27 ± 1.24	0.044
1:1.4	0.67 ± 2.77	4.0	2.121 ± 0.019	3.37 ± 1.21	0.041
1:1.5	0.03 ± 2.10	4.0	2.116 ± 0.014	3.76 ± 0.87	0.048
1:1.6	0.28 ± 3.16	4.0	2.118 ± 0.022	4.51 ± 1.45	0.051
1:1.7	-0.17 ± 3.29	4.0	2.114 ± 0.023	4.54 ± 1.49	0.057
1:1.8	-0.41 ± 3.43	4.0	2.111 ± 0.024	5.12 ± 1.59	0.060
1:1.9	-0.36 ± 3.63	4.0	2.112 ± 0.026	5.04 ± 1.68	0.060
1:2.0	-0.27 ± 3.27	4.0	2.113 ± 0.024	5.52 ± 1.55	0.053

[a]This parameter was fixed during the fit.

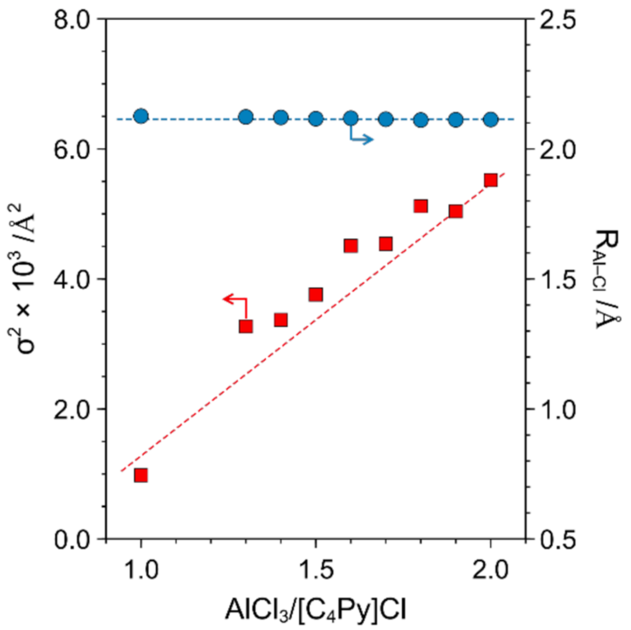

Supplementary Fig. 3 | The corresponding Al–Cl coordination numbers and interatomic distances obtained from EXAFS fit.

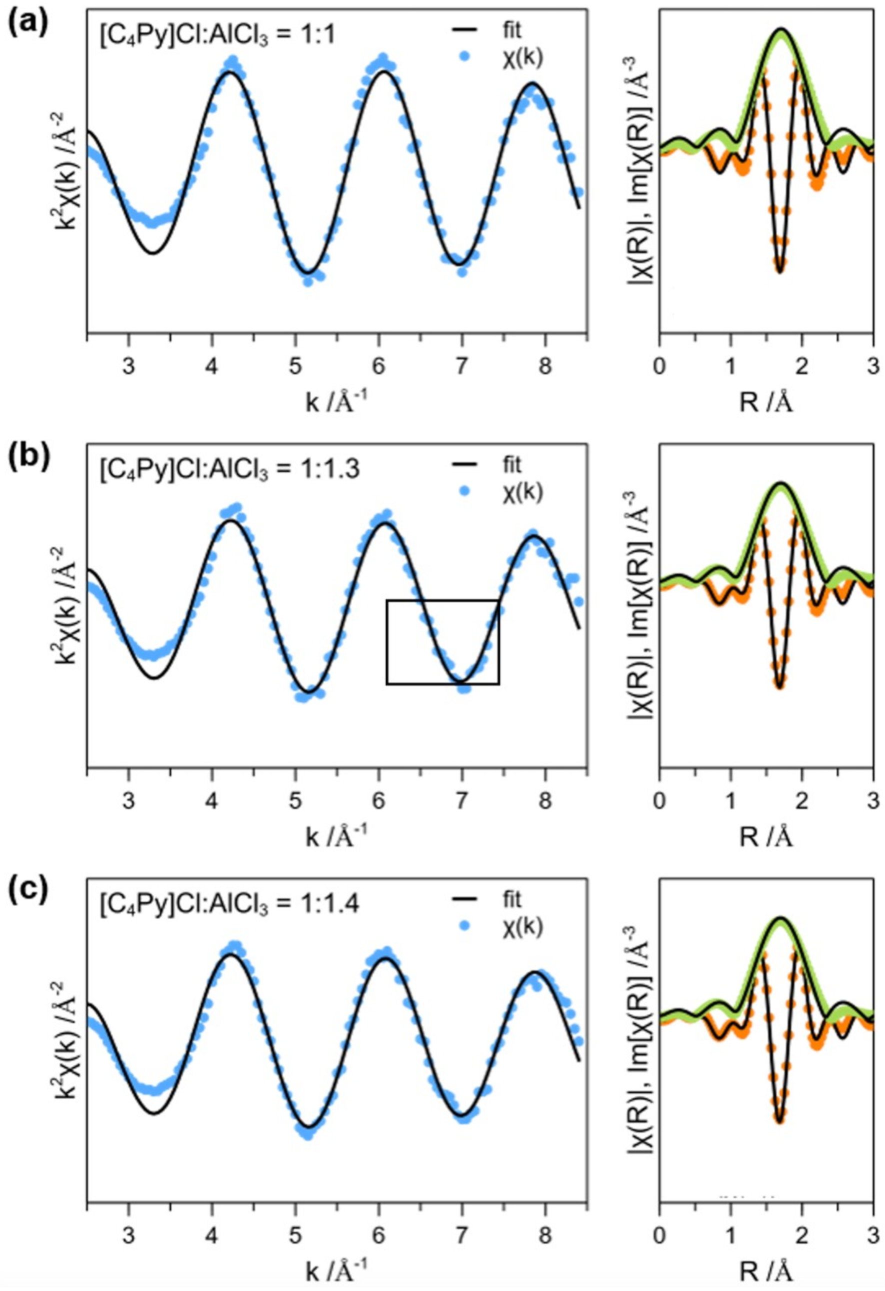

Supplementary Fig. 4 | Al K-edge EXAFS of [C_4Py]Cl-x$AlCl_3$ ionic liquids (x=1, 1.3, and 1.4), where the plots on the left-hand side show the k^2-weighted EXAFS and the plots on the right-hand side show the magnitude (green line) and the imaginary part (orange dots) of the Fourier transform spectra. Experimental data are shown as closed symbols, and the corresponding fits are shown as solid lines. (**a**) [C_4Py]Cl:$AlCl_3$ = 1:1, (**b**) [C_4Py]Cl:$AlCl_3$ = 1:1.3, and (**c**) [C_4Py]Cl:$AlCl_3$ = 1:1.4.

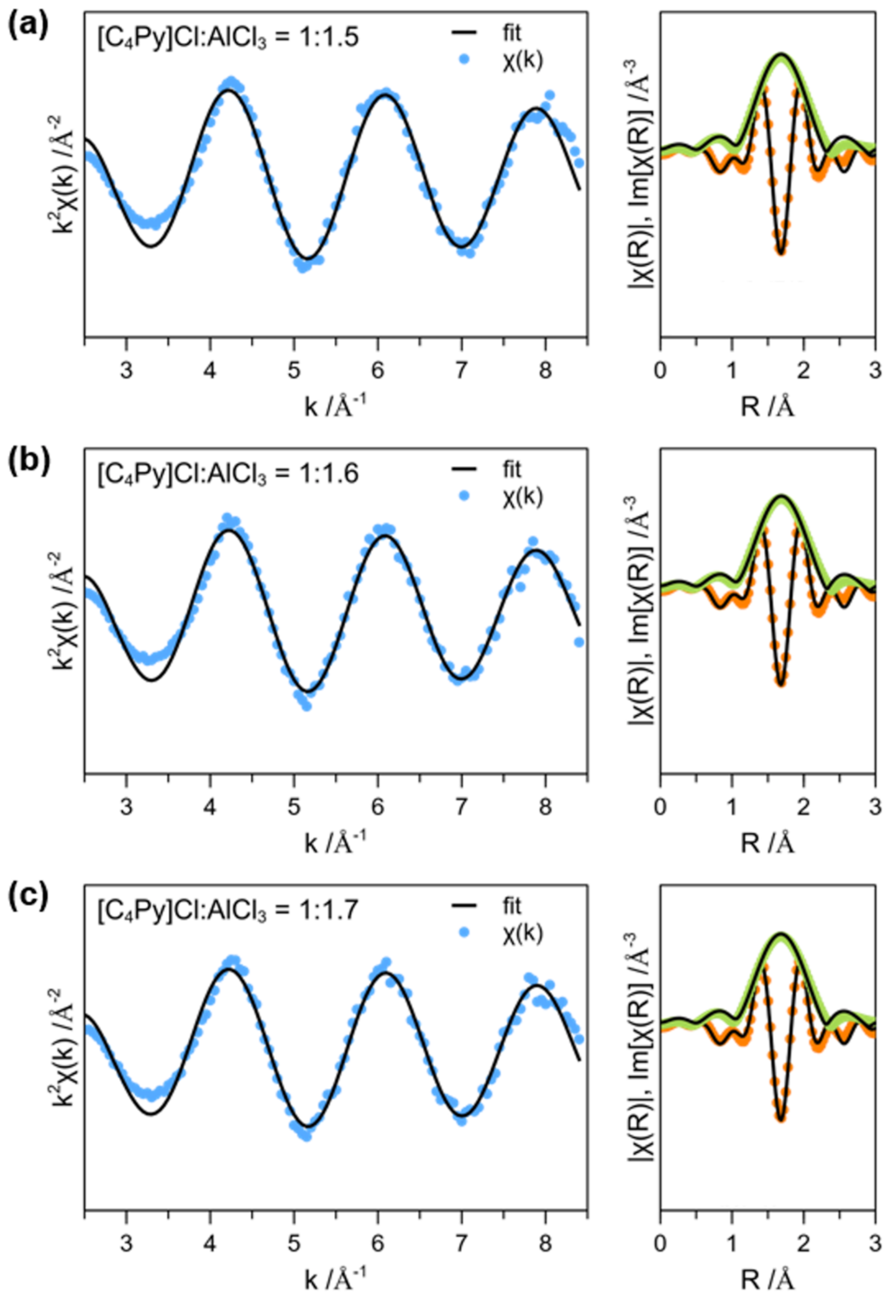

Supplementary Fig. 5 | Al K-edge EXAFS of $[C_4Py]Cl\text{-}xAlCl_3$ ionic liquids (x=1.5, 1.6, and 1.7), where the plots on the left-hand side show the k^2-weighted EXAFS and the plots on the right-hand side show the magnitude (green line) and the imaginary part (orange dots) of the Fourier transform spectra. Experimental data are shown as closed symbols, and the corresponding fits are shown as solid lines. (**a**) $[C_4Py]Cl:AlCl_3$ = 1:1.5, (**b**) $[C_4Py]Cl:AlCl_3$ = 1:1.6, and (**c**) $[C_4Py]Cl:AlCl_3$ = 1:1.7.

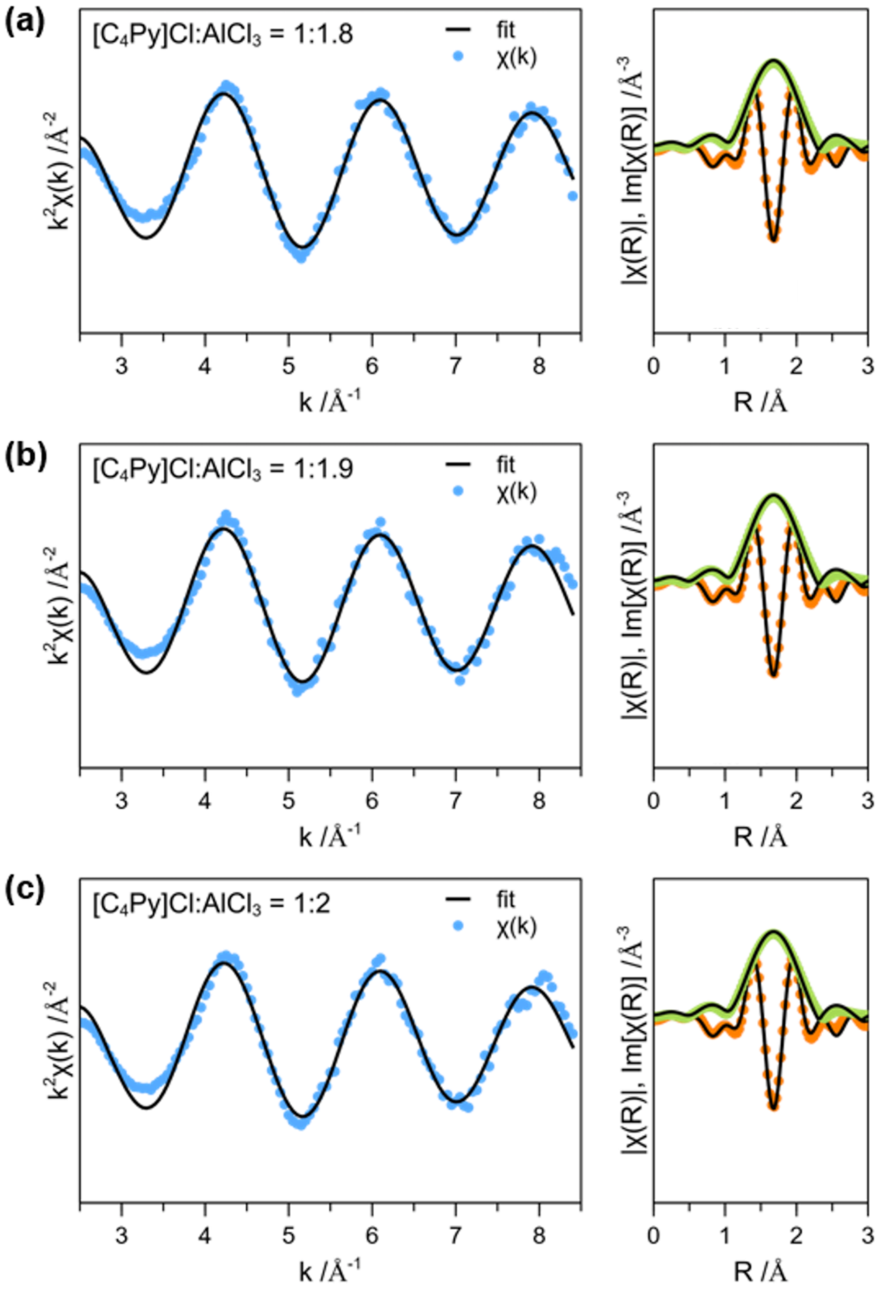

Supplementary Fig. 6 | Al K-edge EXAFS of [C_4Py]Cl-x$AlCl_3$ ionic liquids (x=1.8, 1.9, and 2.0), where the plots on the left-hand side show the k^2-weighted EXAFS and the plots on the right-hand side show the magnitude (green line) and the imaginary part (orange dots) of the Fourier transform spectra. Experimental data are shown as closed symbols, and the corresponding fits are shown as solid lines. (**a**) [C_4Py]Cl:$AlCl_3$ = 1:1.8, (**b**) [C_4Py]Cl:$AlCl_3$ = 1:1.9, and (**c**) [C_4Py]Cl:$AlCl_3$ = 1:2.0.

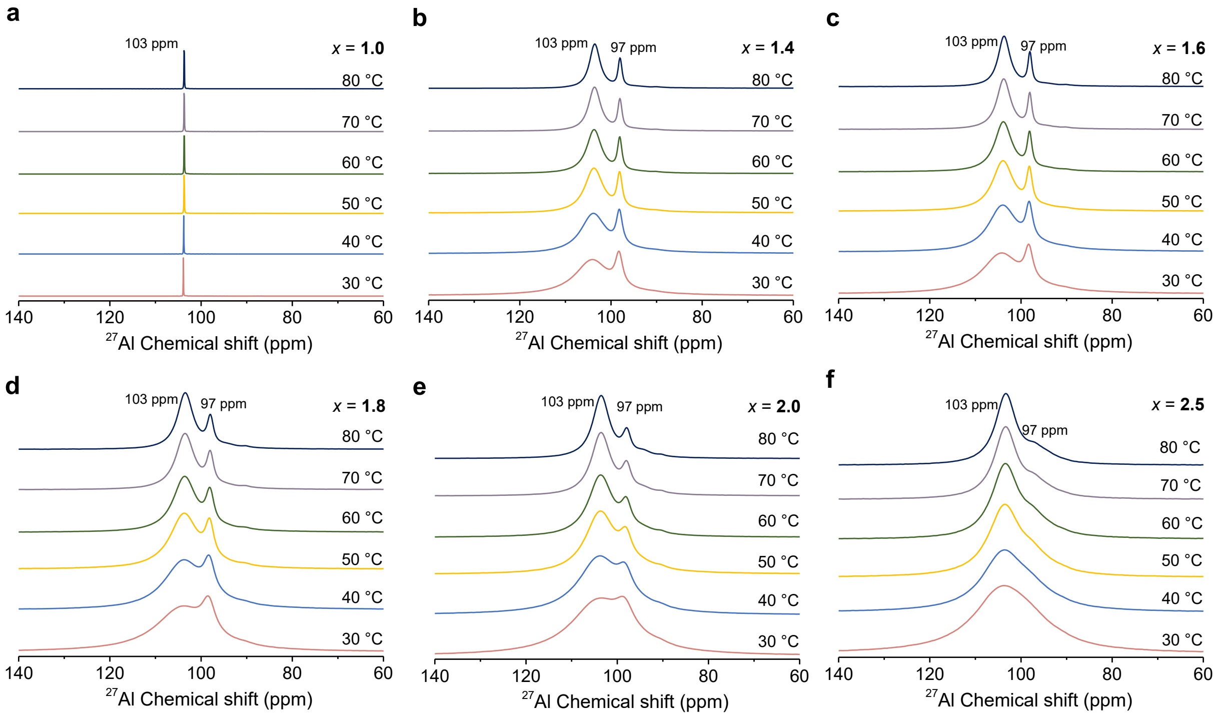

Supplementary Fig. 7 | Temperature-programmed ^{27}Al MAS NMR spectroscopy on chloroaluminate ionic liquids. **a-f**, ^{27}Al MAS NMR spectra of $[C_4Py]Cl$-$x$$AlCl_3$ (x=1-2.5) recorded during temperature-programmed process from 30 °C to 80 °C.

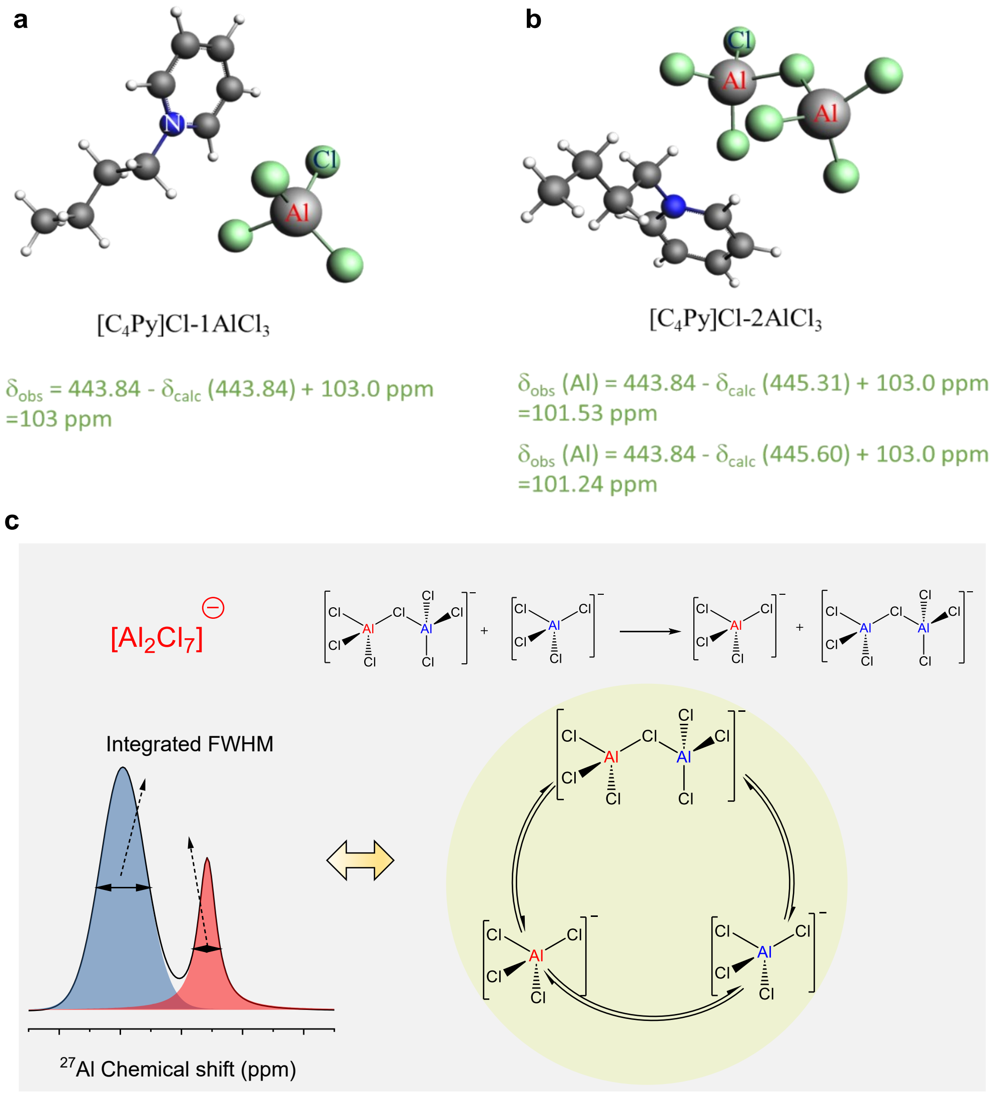

Supplementary Fig. 8 | DFT-NMR modeling of $Al_2Cl_7^-$ showing a broadened linewidth in the ^{27}Al MAS NMR spectra**.** **a**, DFT-NMR modeling of [C_4Py]Cl-*1*$AlCl_3$ and set as reference at 103 ppm. **b**, DFT-NMR modeling of [C_4Py]Cl-2$AlCl_3$ with calculated chemical shift of two Al nuclei. This result highlights the ^{27}Al chemicals shift of $Al_2Cl_7^-$ can be merged into the peak located at around 103 ppm and is upfield shifted by about 2 ppm, thus causing a broadening of the peak at 103 ppm. **c,** Dynamic interconversion between $Al_2Cl_7^-$ and $AlCl_4^-$ via the transient reflected by the integrated linewidths quantified by the full width at half maximum (FWHM).

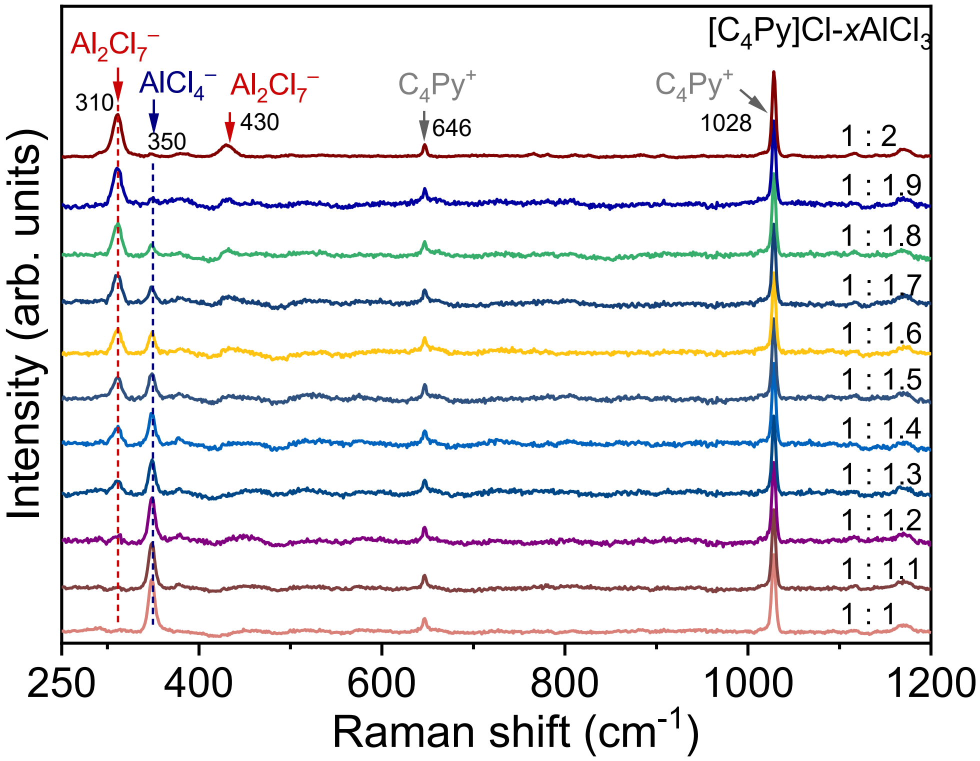

Supplementary Fig. 9 | Raman spectra of [C_4Py]Cl-$x$$AlCl_3$ (x=1-2).

Supplementary Note 2: Derivations of concentration of $AlCl_4^-$ and $Al_2Cl_7^-$ ($Al_2Cl_7^- \rightarrow AlCl_4^- + AlCl_3$) over various time intervals (t)

The chloroaluminate ionic liquids are synthesized by mixing anhydrous aluminium chloride with N-butylpyridinium chloride at various ratios, denoted as [C_4Py]Cl-$x$$AlCl_3$ ($x \in [1,2]$). The formation of [C_4Py]Cl-$x$$AlCl_3$ can simplify as:

$$[C_4Py]Cl + xAlCl_3 \rightarrow [C_4Py]^{\oplus} + (x-1)Al_2Cl_7^{\ominus} + (2-x)AlCl_4^{\ominus} \quad \text{[Rxn S1]}$$

The dissociation of $Al_2Cl_7^{\ominus}$ further generates $AlCl_4^{\ominus}$ and $AlCl_3$-adduct:

$$Al_2Cl_7^{\ominus} \rightarrow AlCl_4^{\ominus} + AlCl_3\ldots\text{adduct} \quad \text{[Rxn S2]}$$

Letting the overall concentrations of $AlCl_3$…adduct be $\sum[AlCl_3 \cdots \text{adduct}]$, then we have:

$$\Delta[AlCl_4^{\ominus}] = \sum[AlCl_3 \cdots adduct] \quad \text{(Eq. S1)}$$

The changes of concentration of $[Al_2Cl_7^{\ominus}]_t$, $[AlCl_4^{\ominus}]_t$ and $\sum[AlCl_3 \cdots \text{adduct}]_t$ at different times can be expressed as:

$$\Delta[Al_2Cl_7^{\ominus}]_t = [Al_2Cl_7^{\ominus}]_0 - [Al_2Cl_7^{\ominus}]_t \quad \text{(Eq. S2)}$$

$$\Delta[AlCl_4^{\ominus}]_t = \sum[AlCl_3 \cdots adduct]_t = [AlCl_4^{\ominus}]_0 + [AlCl_4^{\ominus}]_t \quad \text{(Eq. S3)}$$

$$\frac{[Al_2Cl_7^{\ominus}]_0}{[AlCl_4^{\ominus}]_0} = \frac{x-1}{2-x} \quad \text{(Eq. S4)}$$

$$\frac{[Al_2Cl_7^{\ominus}]_t}{[AlCl_4^{\ominus}]_t} = \frac{[Al_2Cl_7^{\ominus}]_0 - \sum[AlCl_3 \cdots adduct]_t}{[AlCl_4^{\ominus}]_0 + \sum[AlCl_3 \cdots adduct]_t} \quad \text{(Eq. S5)}$$

By substituting with equations S1-S5, the initial concentration of [C_4Py]Cl-$x$$AlCl_3$ was denoted as n_0 mol, then we have:

$$\frac{[Al_2Cl_7^{\ominus}]_t}{[AlCl_4^{\ominus}]_t} = \frac{(x-1)n_0 - \sum[AlCl_3 \cdots adduct]_t}{(2-x)n_0 + \sum[AlCl_3 \cdots adduct]_t} \quad \text{(Eq. S6)}$$

The area ratio of Raman signals at 310 cm^{-1} and 350 cm^{-1} depends closely on the molar ratio of $Al_2Cl_7^{\ominus}$ to $AlCl_4^{\ominus}$[18-2018-2017-19], showing a linear correlation with a constant k_{SCS}, the coefficient:

$$\frac{[Al_2Cl_7^{\ominus}]_t}{[AlCl_4^{\ominus}]_t} = k_{SCS}\left(\frac{Area_{310\ cm^{-1}}}{Area_{350\ cm^{-1}}}\right)_t \quad \text{(Eq. S7)}$$

From Fig. 2e in the main text showing that the $\left(\frac{\mathrm{Area}_{310\ cm^{-1}}}{\mathrm{Area}_{350\ cm^{-1}}+\mathrm{Area}_{310\ cm^{-1}}}\right)_0$ is proportional to *x* (*x* = the molar ratio of $AlCl_3$/[C_4Py]Cl), we have:

$$\left(\frac{Area_{310\ cm^{-1}}}{Area_{350\ cm^{-1}} + Area_{310\ cm^{-1}}}\right)_0 = 1.04x - 1.08 \qquad (R^2 = 0.994) \qquad \text{(Eq. S8)}$$

Based on equations S4, S7 and S8, the k_{SCS} and $\sum[\mathrm{AlCl_3} \cdots \mathrm{adduct}]_t$ can be expressed as:

$$k_{SCS} = \frac{(2.08 - 1.04x)(x-1)}{(2-x)(1.04x - 1.08)} \quad \text{(Eq. S9)}$$

$$\sum[AlCl_3 \cdots adduct]_t = (x-2)n_0 + \frac{n_0}{k_{SCS}\left(\frac{Area_{310\ cm^{-1}}}{Area_{350\ cm^{-1}}}\right)_t + 1} \quad \text{(Eq. S10)}$$

$$[AlCl_4^{\ominus}]_t = (2x-4)n_0 + \frac{n_0}{k_{SCS}\left(\frac{Area_{310\ cm^{-1}}}{Area_{350\ cm^{-1}}}\right)_t + 1} \quad \text{(Eq. S11)}$$

$$[Al_2Cl_7^{\ominus}]_t = n_0 - \frac{n_0}{k_{SCS}\left(\frac{Area_{310\ cm^{-1}}}{Area_{350\ cm^{-1}}}\right)_t + 1} \quad \text{(Eq. S12)}$$

The Al mole fraction (χ_{Al}) of chloroaluminate species can be expressed as:

$$\chi_{Al}(Al_2Cl_7^{\ominus})_t = \frac{2[Al_2Cl_7^{\ominus}]_t}{xn_0};$$
$$\chi_{Al}(AlCl_4^{\ominus})_t = \frac{[AlCl_4^{\ominus}]_t}{xn_0}; \quad \text{(Eq. S13)}$$
$$\chi_{Al}\left(\sum[AlCl_3 \cdots adduct]\right)_t = \frac{\sum[AlCl_3 \cdots adduct]_t}{xn_0}$$

We then plotted the k_{SCS} against x using equation S8, see below:

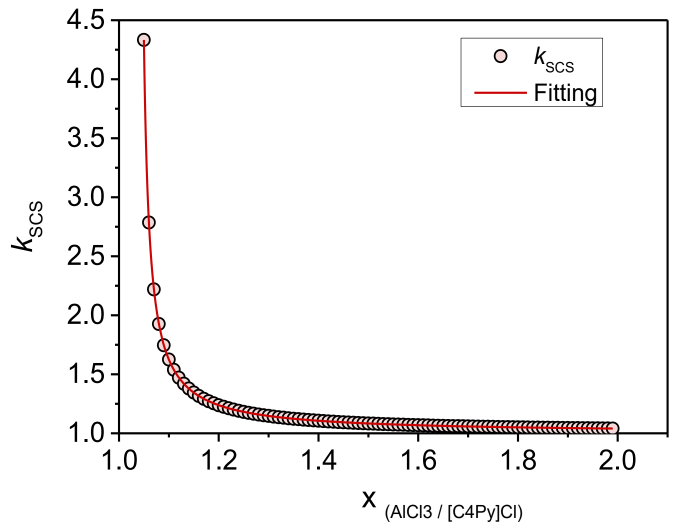

❖ **For [C$_4$Py]Cl-2AlCl$_3$ (x=2)**

$\lim_{x\to 2} k_{\mathrm{SCS}} = 1$, the concentration of chloroaluminate species can be rewritten as:

$$[AlCl_4{}^{\ominus}]_t = \frac{n_0}{\left(\frac{Area_{310\ cm^{-1}}}{Area_{350\ cm^{-1}}}\right)_t + 1} \quad \text{(Eq. S14)}$$

$$[Al_2Cl_7{}^{\ominus}]_t = n_0 - \frac{n_0}{\left(\frac{Area_{310\ cm^{-1}}}{Area_{350\ cm^{-1}}}\right)_t + 1} \quad \text{(Eq. S15)}$$

The Al mole fraction (χ_{Al}) of chloroaluminate species can be expressed as:

$$\chi_{Al}(\sum[Al - adduct])_t = \chi_{Al}\left(AlCl_4{}^{\ominus}\right)_t = \frac{1}{2\left(\frac{Area_{310\ cm^{-1}}}{Area_{350\ cm^{-1}}}\right)_t + 2} \quad \text{(Eq. S16)}$$

$$\chi_{Al}\left(Al_2Cl_7{}^{\ominus}\right)_t = 1 - \frac{1}{\left(\frac{Area_{310\ cm^{-1}}}{Area_{350\ cm^{-1}}}\right)_t + 1} \quad \text{(Eq. S17)}$$

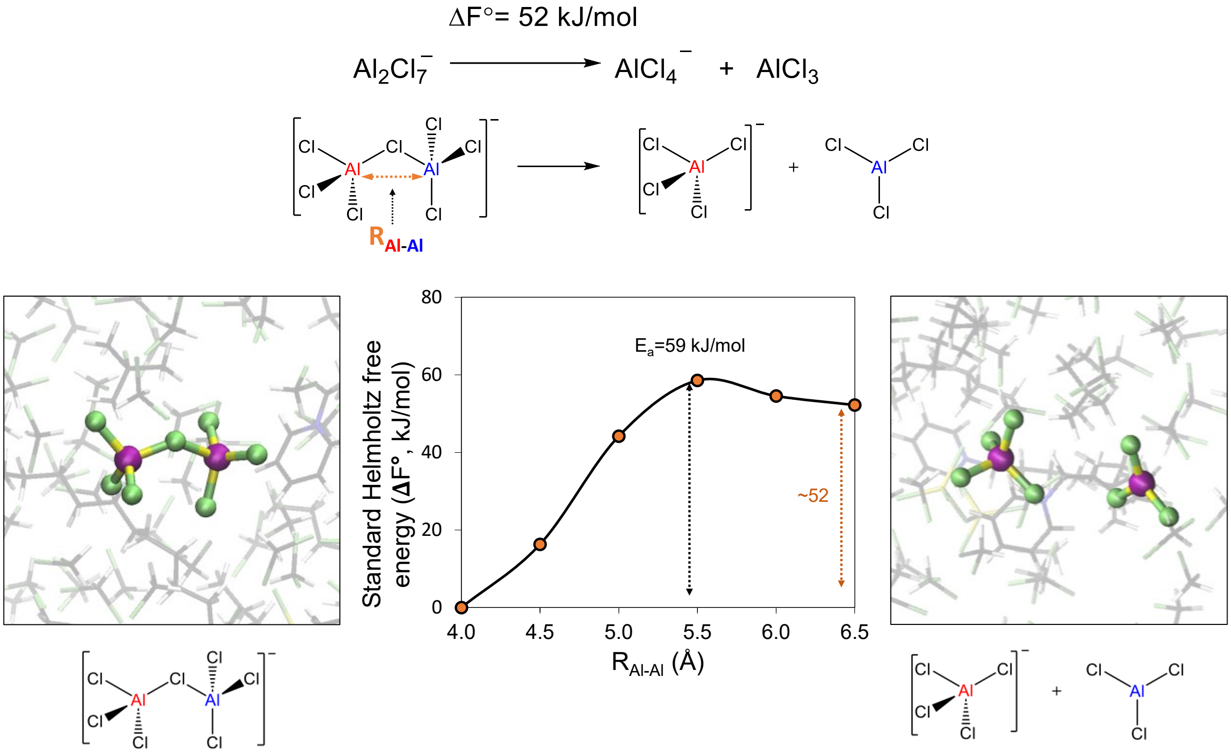

Supplementary Fig. 10 | The computational simulation of the dissociation of $Al_2Cl_7^-$ in a DCM solvent. The standard Helmholtz free energies (ΔF°) as a function of the Al distance between two Al center of $Al_2Cl_7^-$ (These data were acquired through ab initio molecular dynamics simulations in conjunction with the Blue Moon ensemble method).

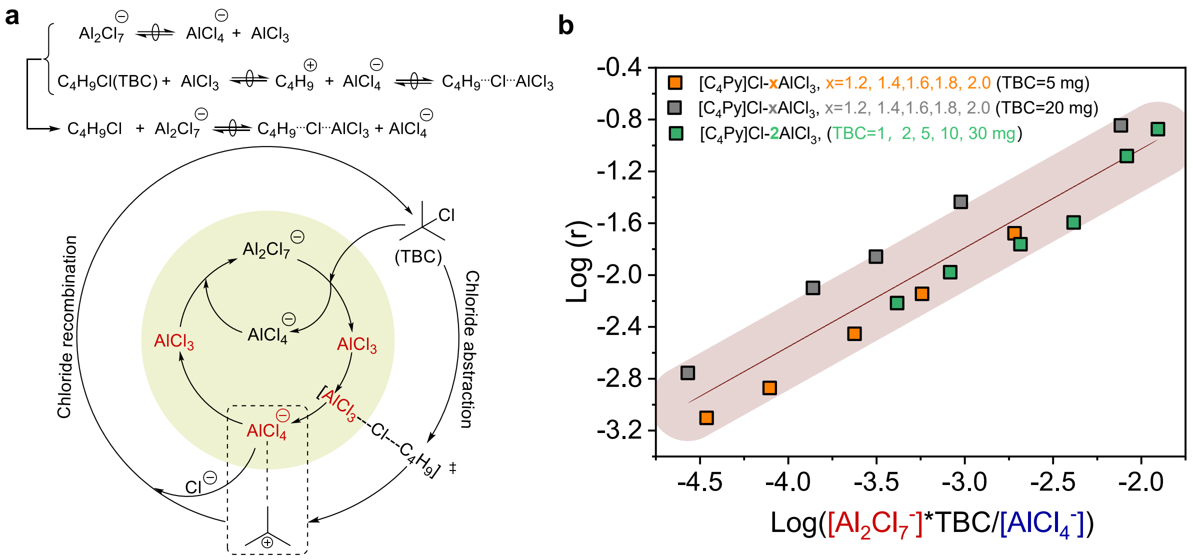

Supplementary Fig. 11 | **a**, Illustration of chloroaluminate transformation in the presence of TBC The initial reaction rate as a function of TBC concentration . **b**, The initial LDPE conversion rate as a function of $\frac{[Al_2Cl_7]^-[TBC]}{[AlCl_4]^-}$concentration**.** The concentration of $\frac{[Al_2Cl_7]^-[TBC]}{[AlCl_4]^-}$was determined by varying the molar ratio of initial anhydrous $AlCl_3$ and [C_4Py]Cl, and TBC amount.

Note that: the initial rates calculated as grams of LDPE converted per hour and per mole of Al content. The concentration of $AlCl_3$-adducts ($AlCl_3$···Cl···C_4) can be expressed as: $[AlCl_3\cdots Cl\cdots C4] = \frac{[Al_2Cl_7]^-*[TBC]}{[AlCl_4]^-*K}$. The plot of Log(r) *vs*. Log($\frac{[Al_2Cl_7]^-[TBC]}{[AlCl_4]^-}$) showed a nearly follow first order kinetics. The initial reaction rate is directly proportional to the concentration of TBC loading.

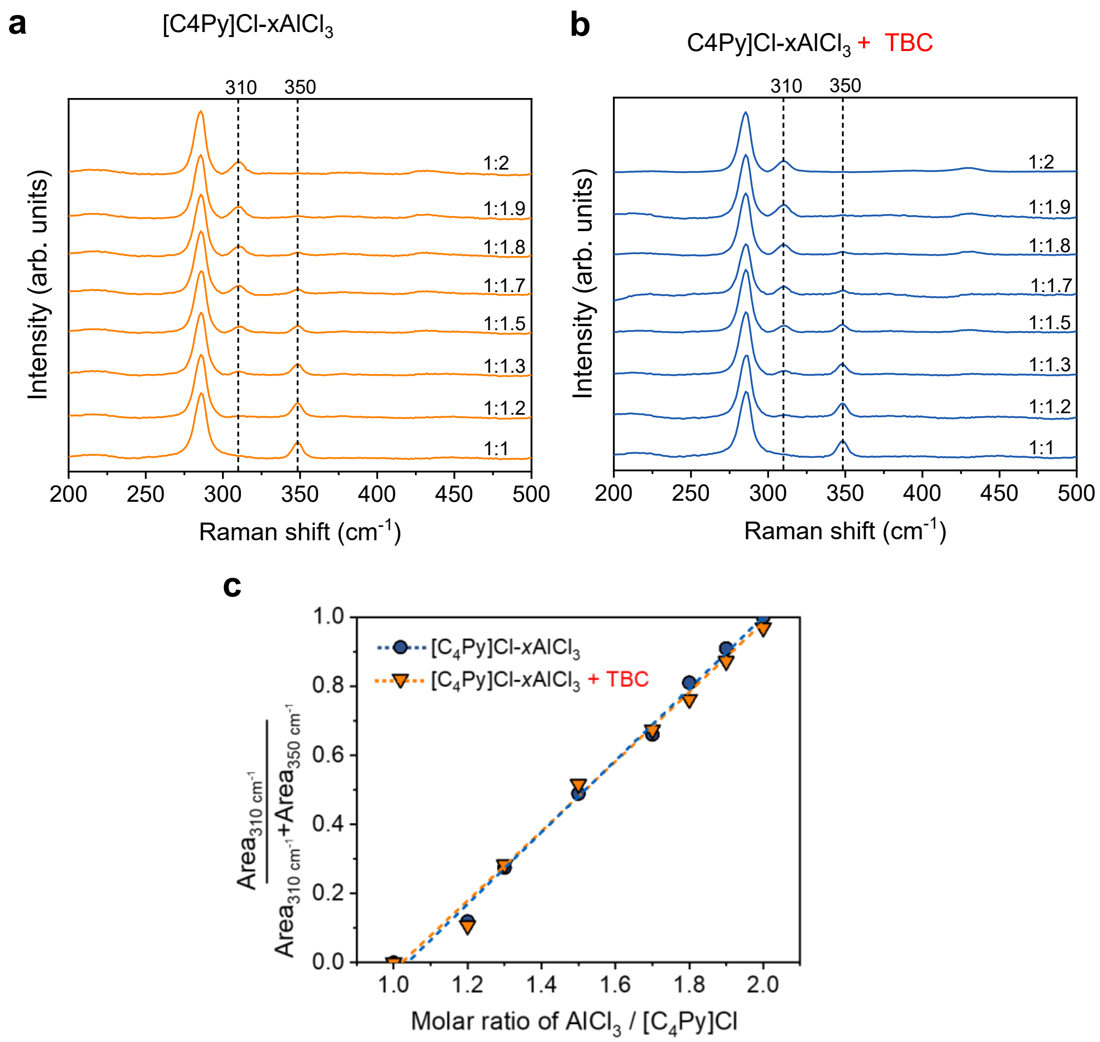

Supplementary Fig. 12 | Raman spectroscopy analysis of the interaction between TBC and $[C_4Py]Cl\text{-}xAlCl_3$. **a**, Raman spectra of neat $[C_4Py]Cl\text{-}xAlCl_3$ ionic liquids. **b**. Raman spectra of $[C_4Py]Cl\text{-}xAlCl_3$ ionic liquids in the presence of TBC. Spectra recorded at room temperature with TBC to $[C_4Py]Cl\text{-}xAlCl_3$ molar ratio of 1/10. **c**. Plot of the Raman area ratio of $Al_2Cl_7^-$ versus the molar ratio of $AlCl_3/[C_4Py]Cl$ (x).

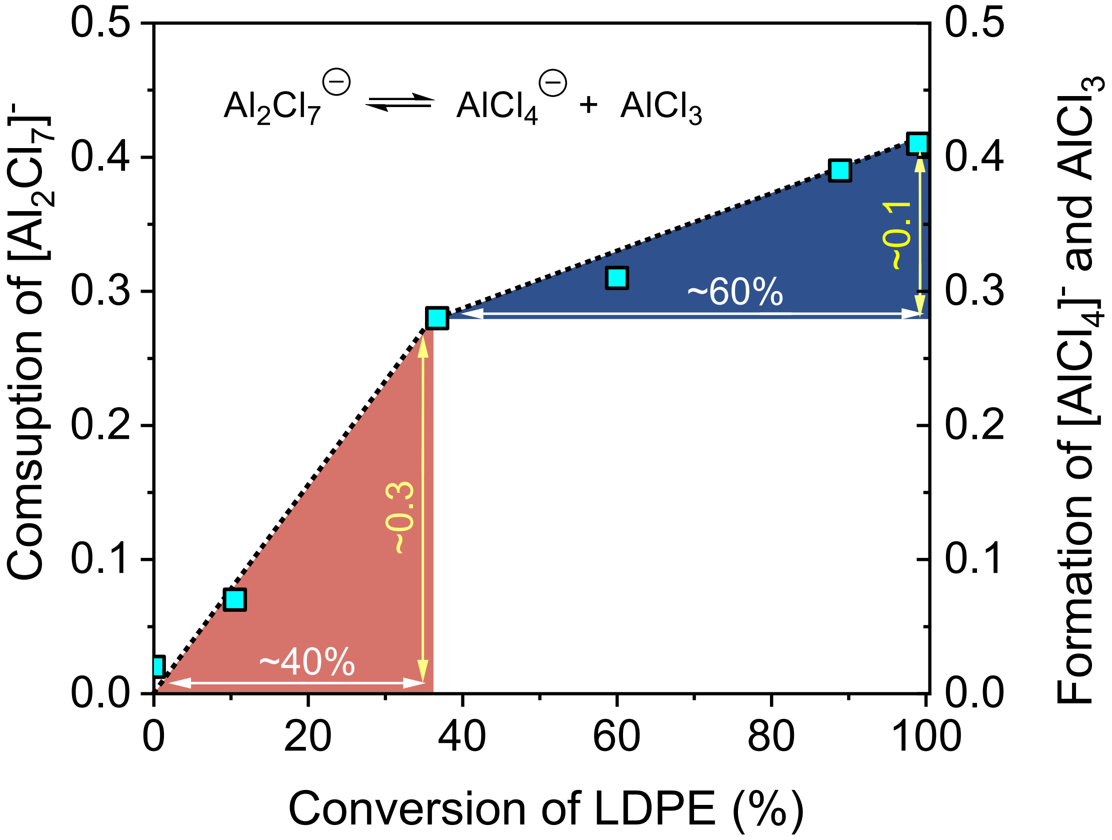

Supplementary Fig. 13 | The conversion of LDPE plotted against the corresponding variation of chloroaluminate species in ionic liquids. The corresponding data are derived from the Raman spectra in Fig. 4b in main text.

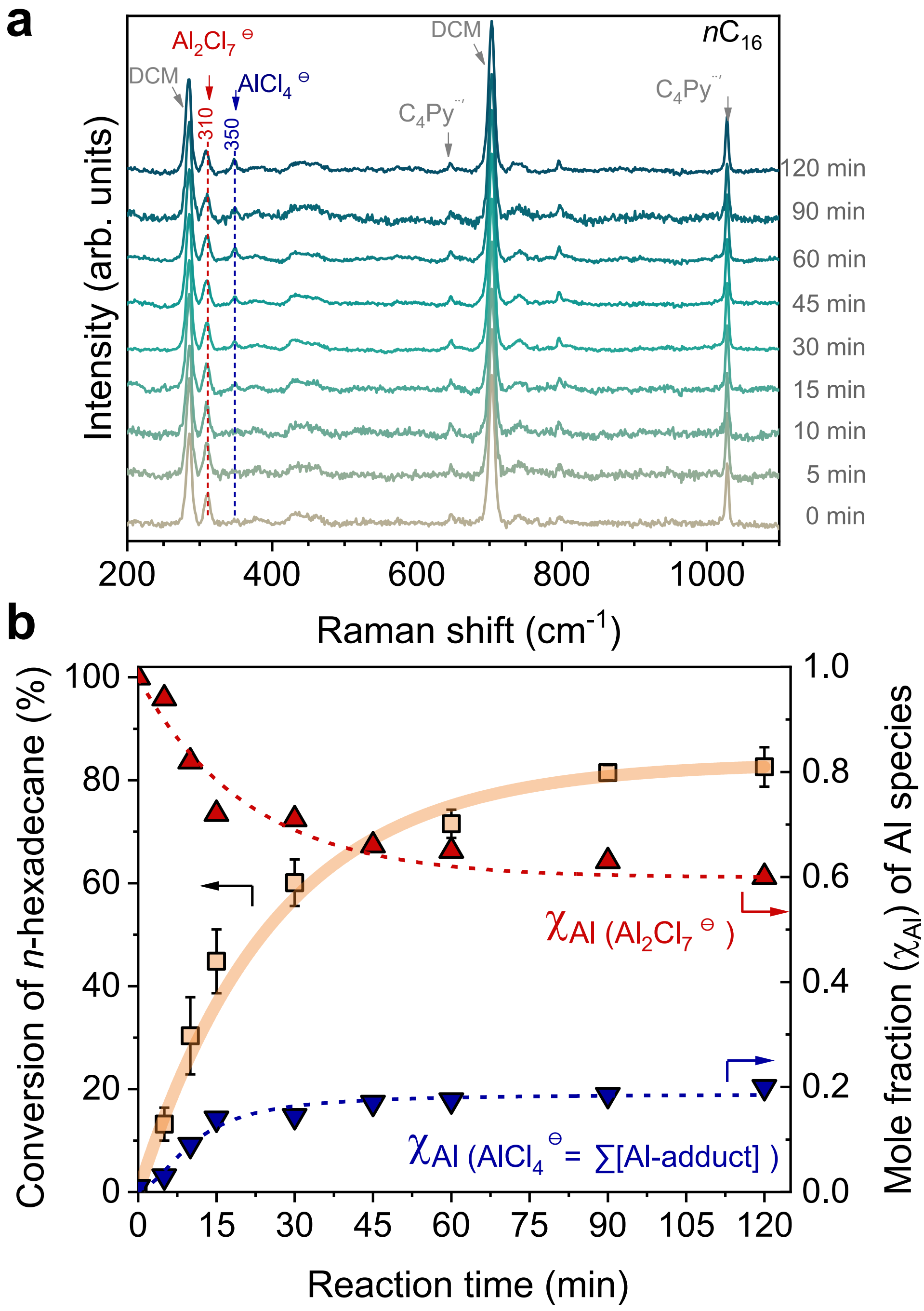

Supplementary Fig. 14 | a, In situ Raman spectra recorded during the cracking-alkylation of *n*-$C_{16}H_{34}$ and iC$_5$ in the presence of TBC additive. **b**, The time-resolved conversion profile of *n*-$C_{16}H_{34}$ and the corresponding variation of chloroaluminate species in ionic liquids. Conditions were as follows: **a-b**: *n*-$C_{16}H_{34}$, 200mg; *i*C$_5$, 800 mg; [C$_4$Py]Cl-2AlCl$_3$, 2 mmol; TBC, 5 mg; DCM, 3 ml; and temperature, 70 °C.

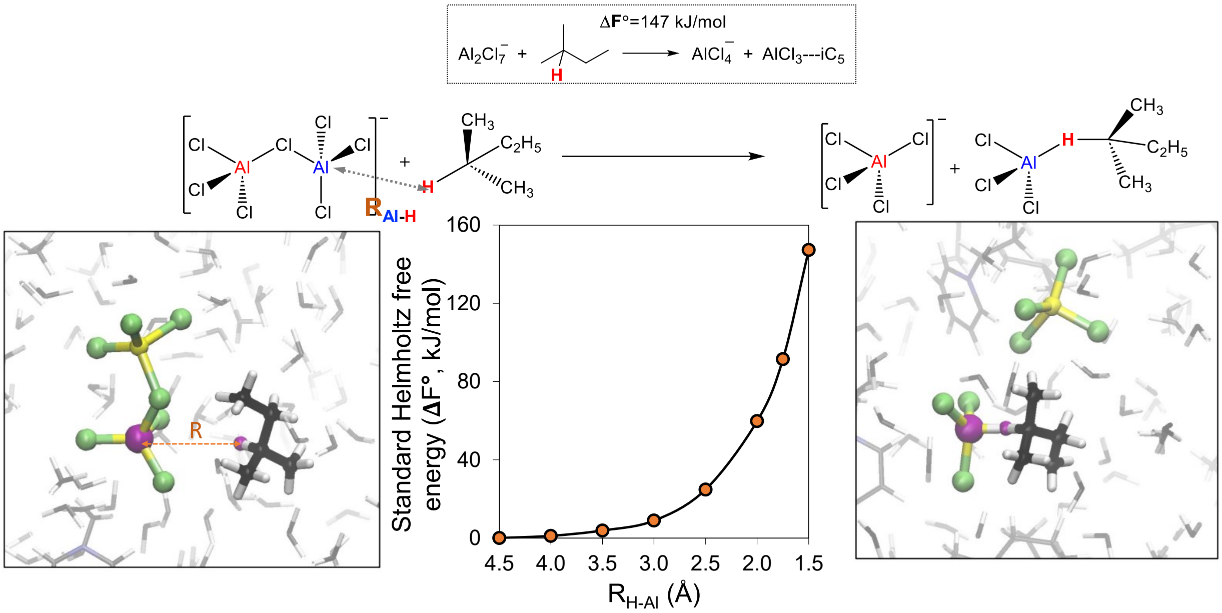

Supplementary Fig. 15 | The computational simulations of $Al_2Cl_7^-$ catalyzed hydride abstraction from *i*C_5. The standard Helmholtz free energies (ΔF°) as a function of the Al–H distance ($R_{Al–H}$) between two molecules, accompanied by atomic configurations that depict the initial and intermediate states. These data were derived through ab initio molecular dynamics simulations in conjunction with the Blue Moon ensemble method.

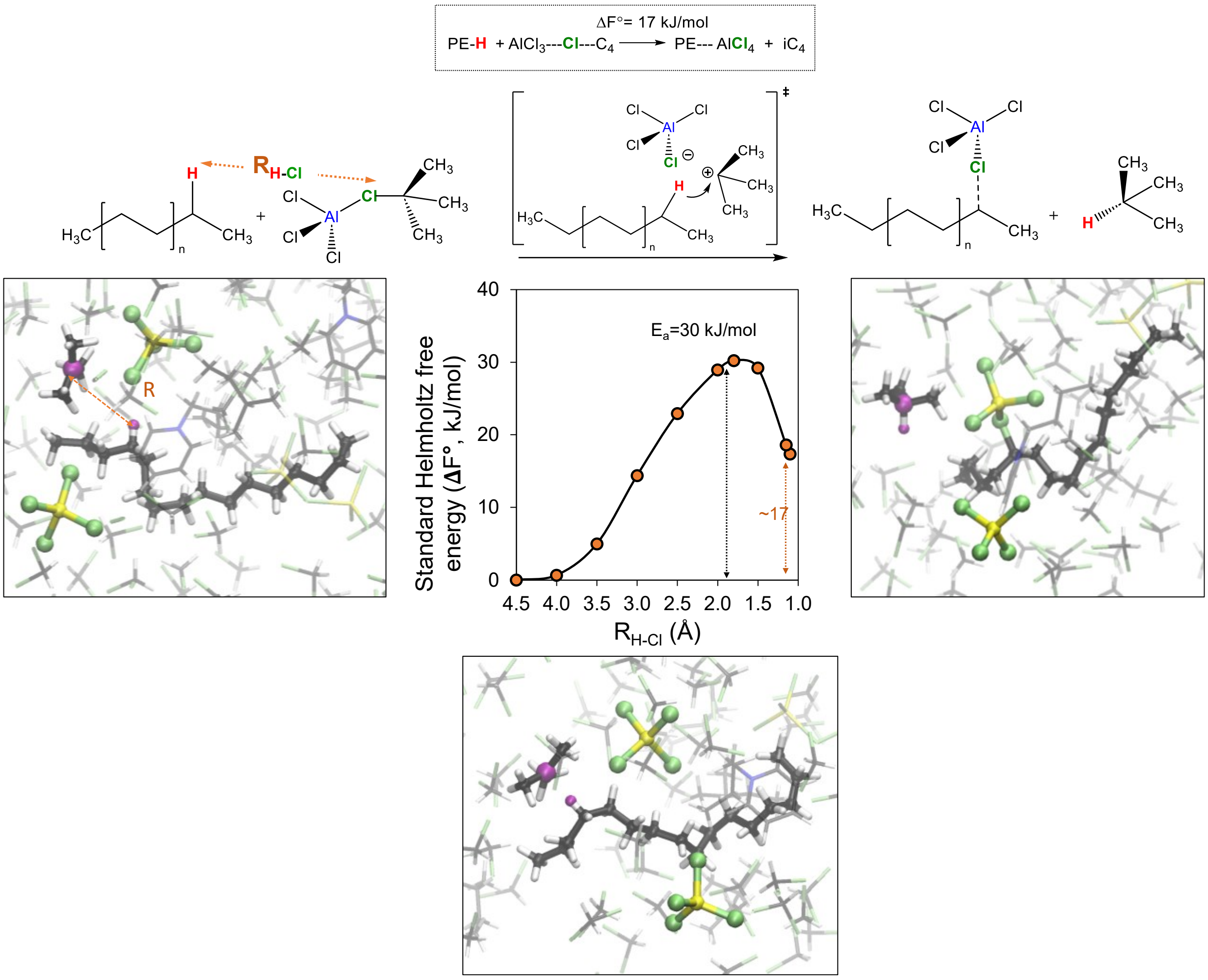

Supplementary Fig. 16 | Computational simulation of $AlCl_3$-TBC adduct catalyzing hydride abstraction from nC_{16}. The standard Helmholtz free energies (ΔF°), dependent on the H–Cl distance ($R_{H\text{–}Cl}$) between two molecules, are accompanied by atomic configurations that represent the initial, transition, and intermediate states. These data were acquired through ab initio molecular dynamics simulations in conjunction with the Blue Moon ensemble method.

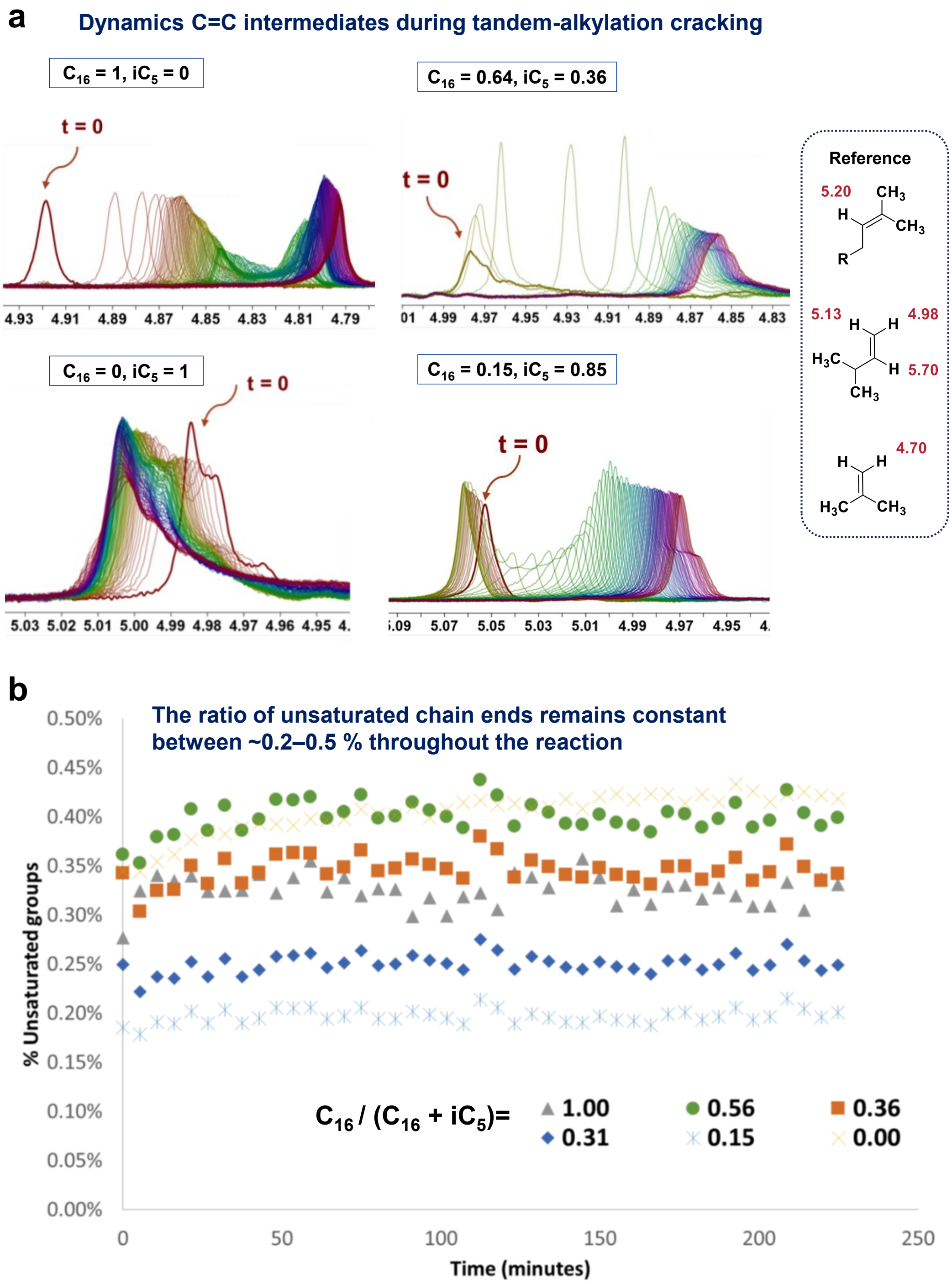

Supplementary Fig. 17 | In situ 1H NMR spectra of tandem cracking-alkylation of n-C_{16} and iC_5 over $[C_4Py]Cl\text{-}2AlCl_3$. **a**, The representative in situ ^1H NMR spectra varying n-C_{16} and iC_5 loading. **b**, the fraction of total alkenes correlates with time. Conditions: Hexadecane (n-C_{16}) 0.16 mmol, isopentane (*i*C_5) 0-0.90 mmol, $C_4Py]Cl\text{-}2AlCl_3$ 0.23 mmol, TBC 0.5 µmol, CD_2Cl_2, 25 °C, 4 h.

Note: For a typical experiment, an NMR tube was charged with $[C_4PyCl]\text{-}2AlCl_3$ (0.23 mmol), C_{16} (0.16 mmol or 2.5 mmol C), and TBC (0.5 µmol). Varying amounts of iC_5 were added under the standard reaction conditions previously described to investigate the impact of the alkylating

agent on alkene detection. The variation in the number of unsaturated hydrogen atoms within the olefinic region (4.4–6.5 ppm). This variability indicates the presence and fluctuation of olefinic intermediates throughout the reaction. The ratio of unsaturated chains remains constant, maintaining a low concentration approximately between 0.2-0.5% throughout the reaction.

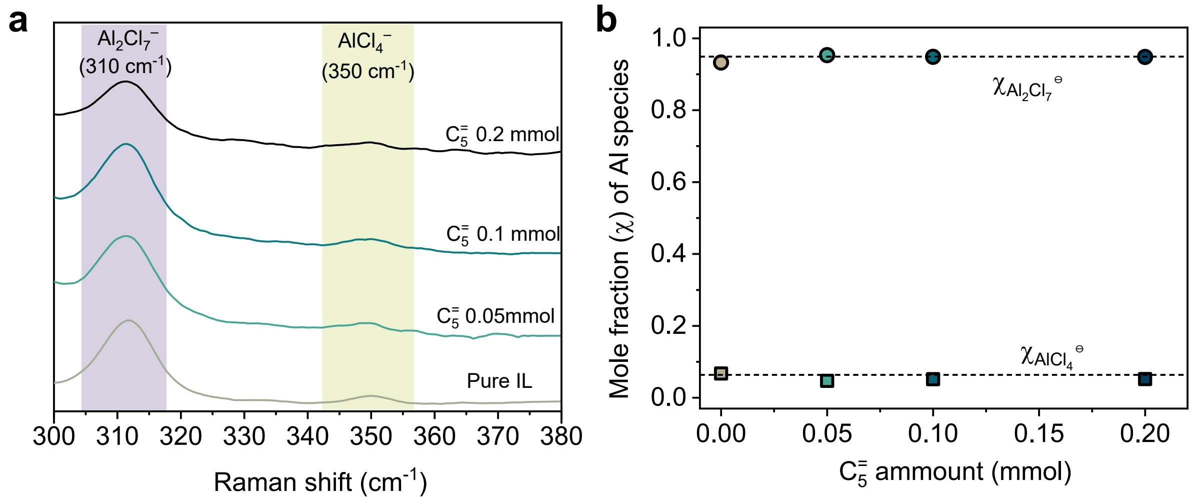

Supplementary Fig. 18 | Raman spectroscopy analysis of the interaction between TBC and [C_4Py]Cl-2$AlCl_3$. **a**, Raman spectra of [C_4PyCl]-2$AlCl_3$ ionic liquids in the presence of pentene ($C_5^=$) and (b) The corresponding variation of chloroaluminate species. Note: spectra recorded at room temperature with varying $C_5^=$ amount (0-0.2mmol) to 2 mmol [C_4PyCl]-2$AlCl_3$ ionic liquids.

The spectra of [C_4Py]Cl-2$AlCl_3$ remained virtually unchanged in the presence of varying concentrations of pentene ($C_5^=$) as well as in its absence. This observation implies that olefin incorporation does not promote the dissociation of $Al_2Cl_7^-$.

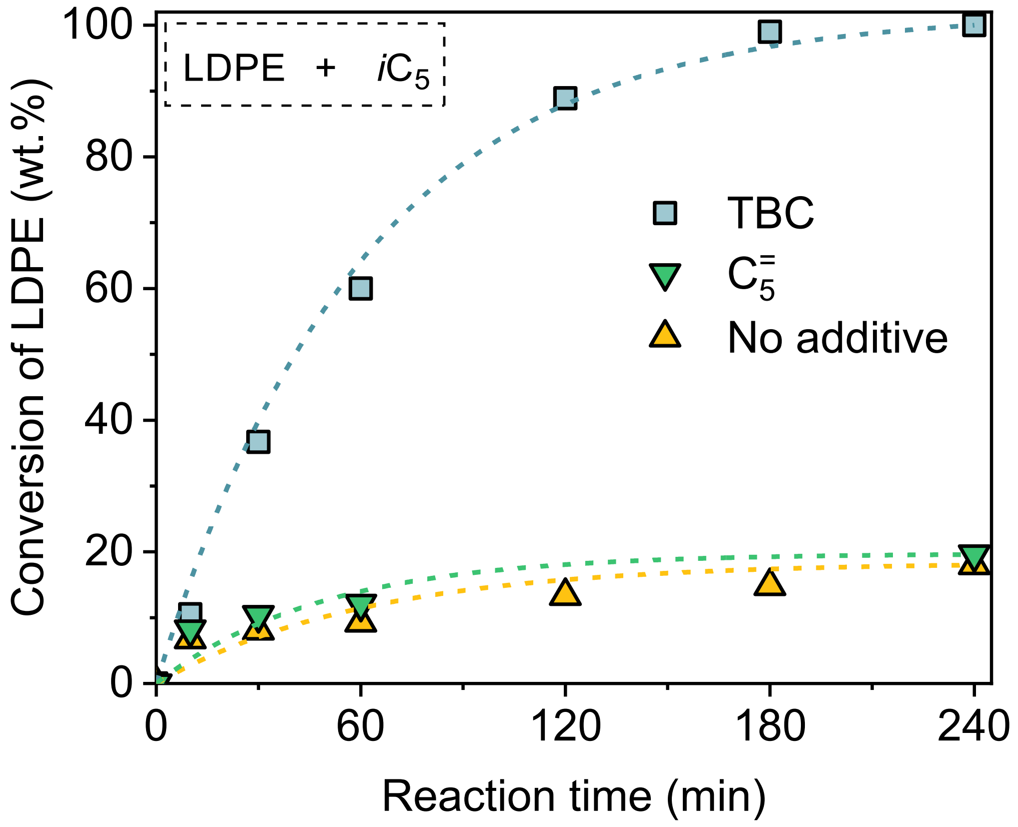

Supplementary Fig. 19 | Time-resolved conversion profile of LDPE in the presence of TBC or $C_5^=$ as additives, compared to conditions without additives. Reaction conditions: LDPE 200 mg, iC_5 800 mg, $[C_4Py]Cl\text{-}AlCl_3$ 3 mmol, additive 0.05 mmol, DCM 3 mL, 70 °C.

Note: the introduction of $C_5^=$ gave only 18 wt.% conversion to LDPE, exhibiting no discernible enhancement when compared to the baseline conditions that lacked such additives. In contrast, the addition of TBC significantly increased the initial reaction rate, resulting in 100% of LDPE conversion within 180 mins (3h). Obviously, $C_5^=$ exhibits a pronounced preference for alkylation with i.

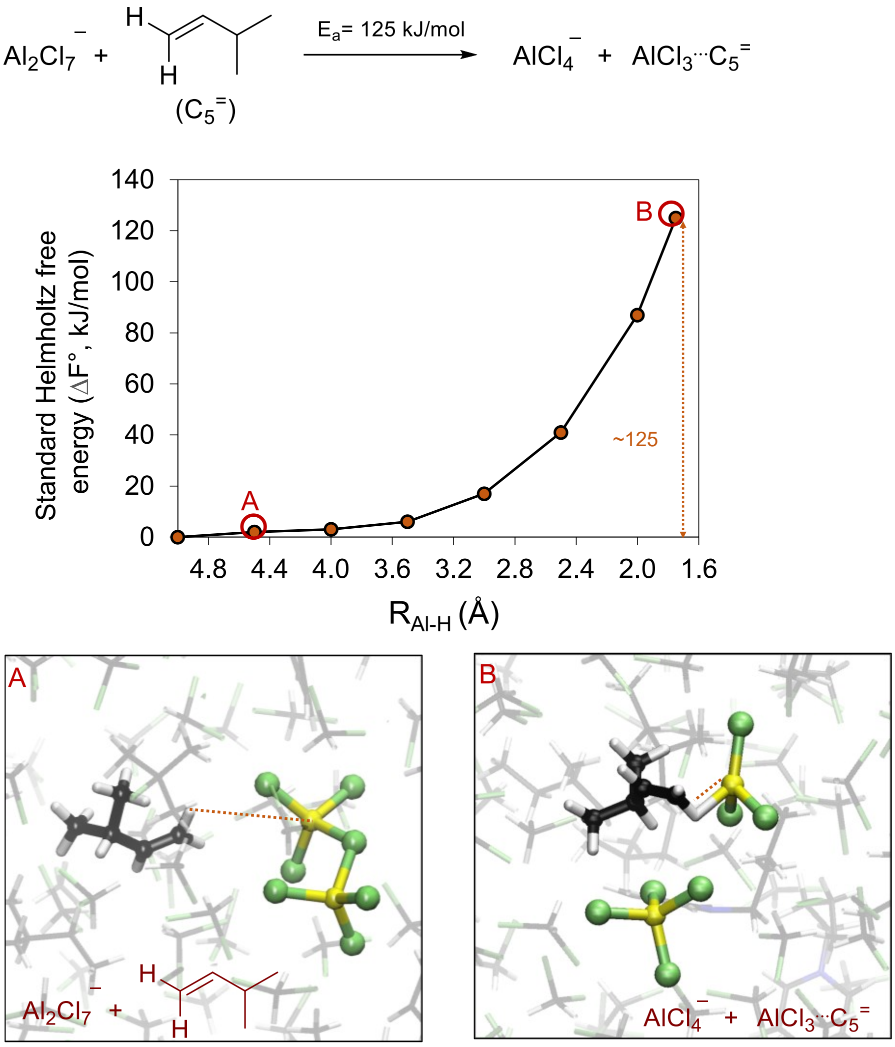

Supplementary Fig. 20 | Computed reaction pathway for the formation of $AlCl_3$-$C_5^=$ adduct. Plotted is the standard Helmholtz free energy (ΔF°) as a function of the internuclear distance of Al-H for the reaction of $Al_2Cl_7^-$ and $C_5^=$ to form $AlCl_4^-$ and a $AlCl_3$-$C_5^=$ adduct. ΔF° is calculated using the Blue Moon ensemble approach for ab initio molecular dynamics. Included are representative structures along the reaction pathway.

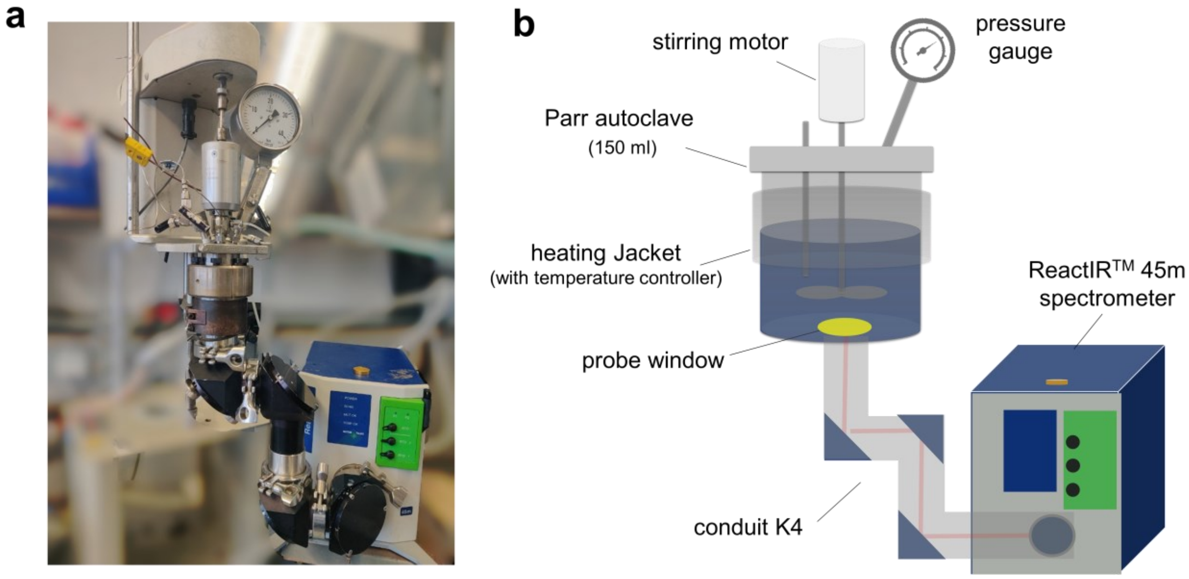

Supplementary Fig. 21 | a, Photograph of the Operando IR setup combing a ReactIRTM 45m spectrometer connected to a 150 mL Parr autoclave, with a probe window connected to the sentinel probe through the conduit K4 (Photo: W. Zhang/ Lercher's Lab in TU Munich). **b**, Schematic illustration of the corresponding Operando IR setup and autoclave reactor.

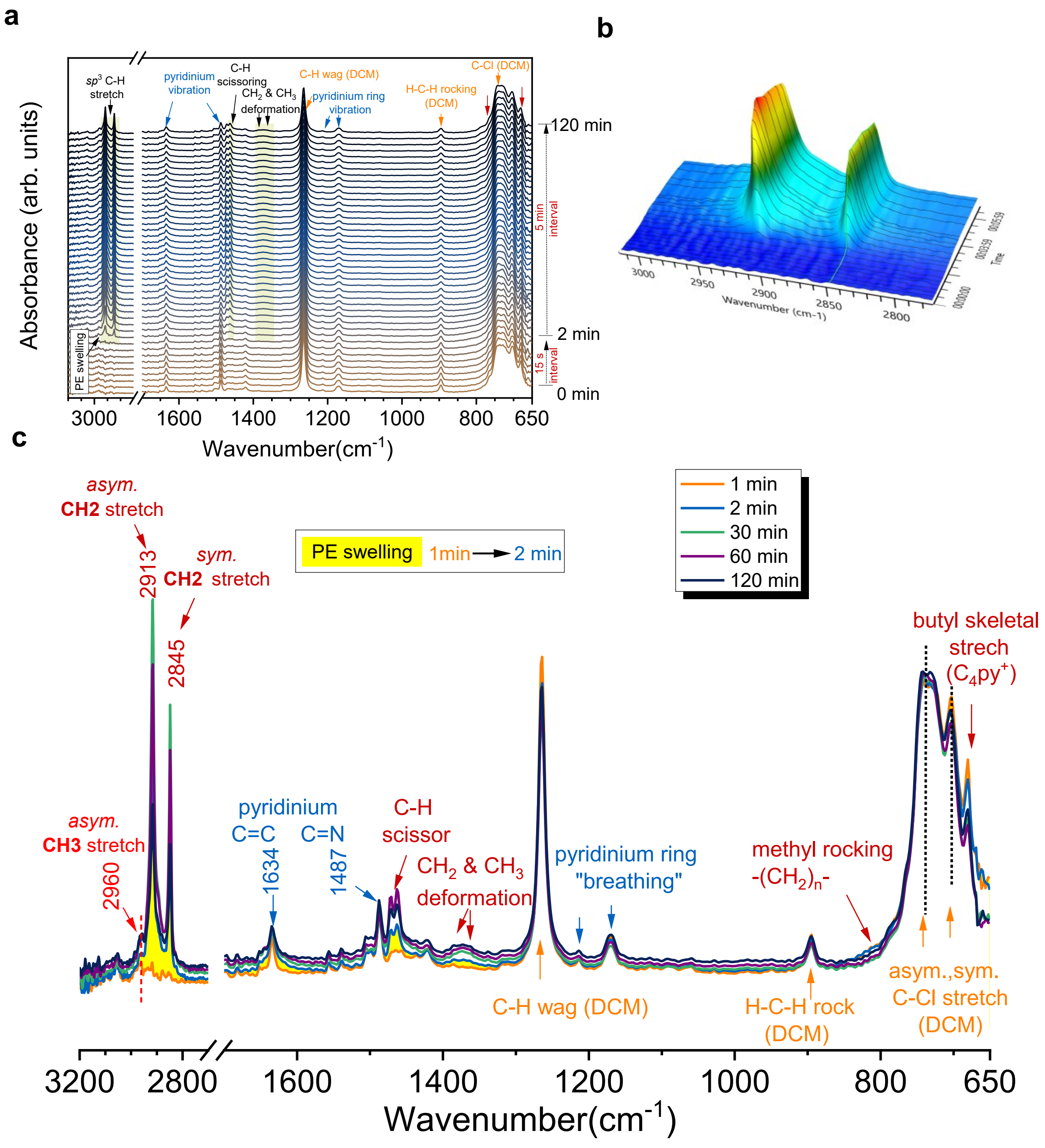

Supplementary Fig. 22 | Operando IR spectra were recorded during LDPE depolymerization in the absence of iC5 over ionic liquids ([C4Py]Cl-2AlCl3). **a**, The time-resolved full spectra, Data were acquired with an automatic sample scan interval of 15s over the range of 3200-650 cm^{-1}, and only presented the selected data with an interval of 5 min. **b,** Three-dimensional spectra of the *sp*^{3} C–H vibrations. **c**, the selected IR spectra were recorded during LDPE depolymerization in the presence of *i*C5 over ionic liquids ([C4Py]Cl-2AlCl3) at 1, 2, 30, 60 and 120 min. Conditions were as follows: LDPE, 2 g; DCM, 30 ml; [C4Py]Cl-2AlCl3, 20 mmol; TBC 0.5 mmol, and temperature, 60 °C.

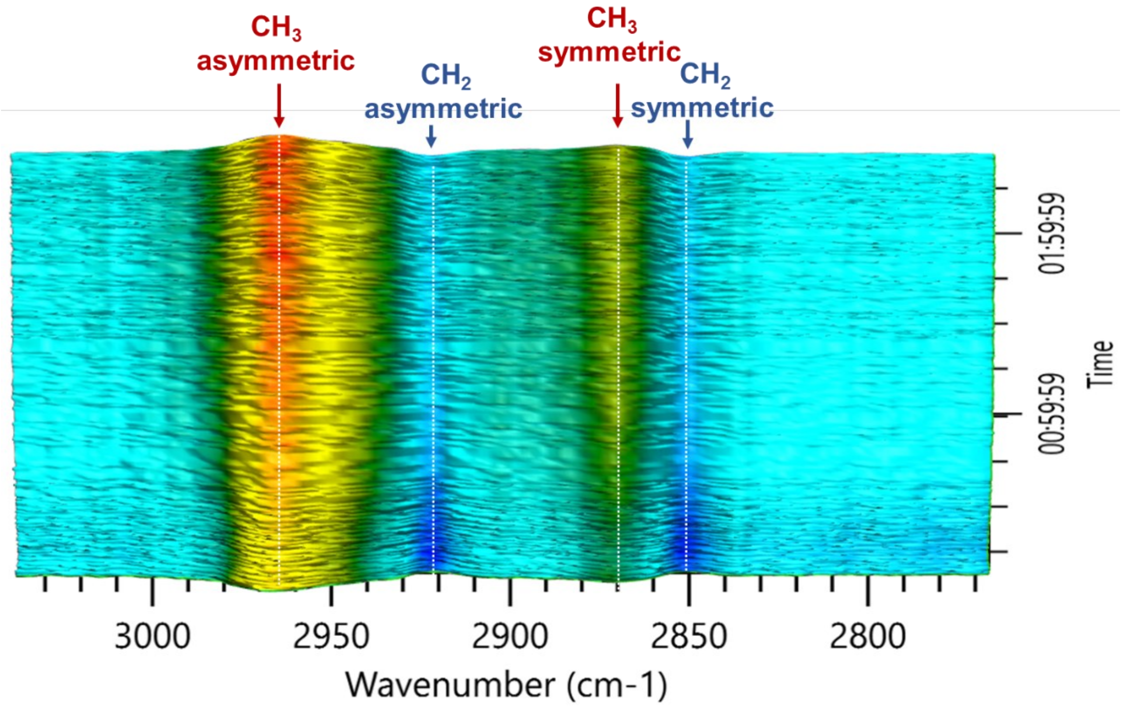

Supplementary Fig. 23 | Three-dimensional operando IR spectra of the *sp*^{3} C–H vibrations as a function of reaction time, recorded during LDPE depolymerization in the presence of iC_5 over ionic liquids ([C_4Py]Cl-2AlCl_3) at 60 °C. The corresponding full spectra are shown in Fig. 5 in the main text.

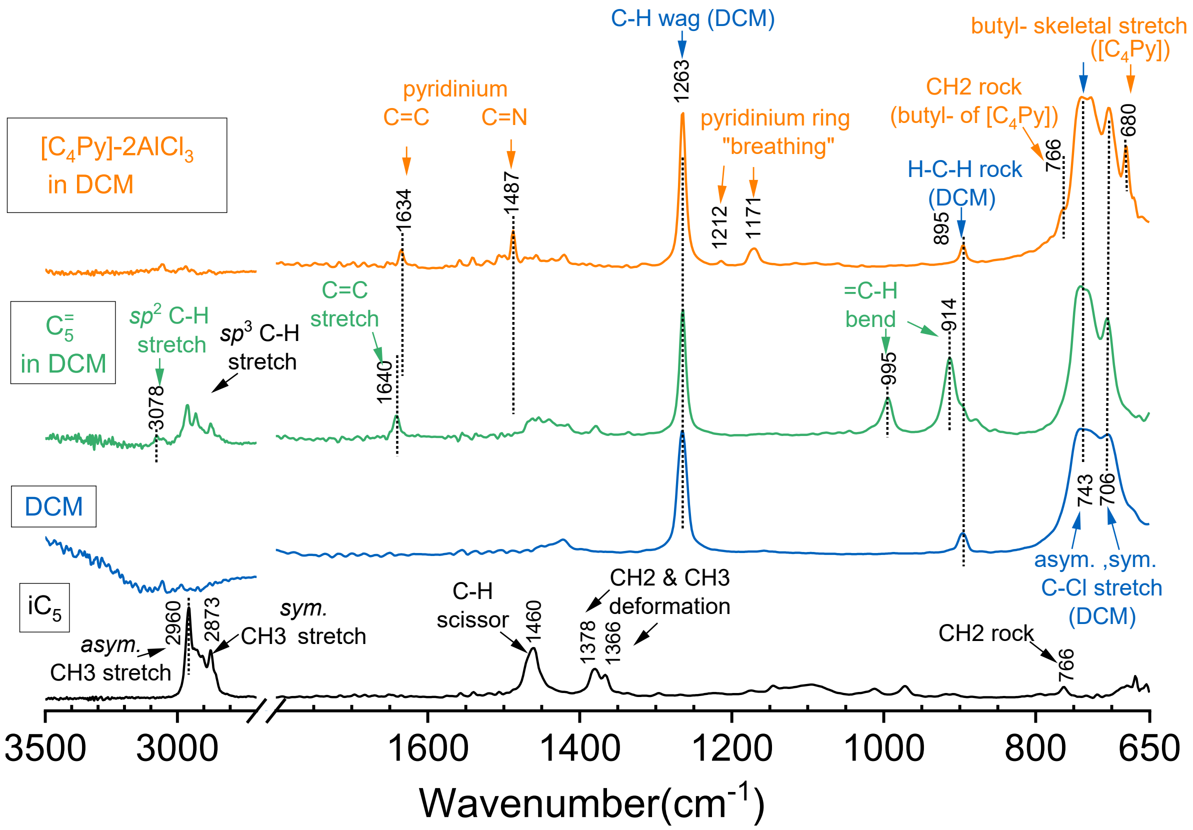

Supplementary Fig. 24 | Reference IR spectra of pure compounds including isopentane (iC_5), Dichloromethane (DCM), 1-pentene ($C_5^=$) and [C_4Py]Cl-2$AlCl_3$ in DCM, respectively.

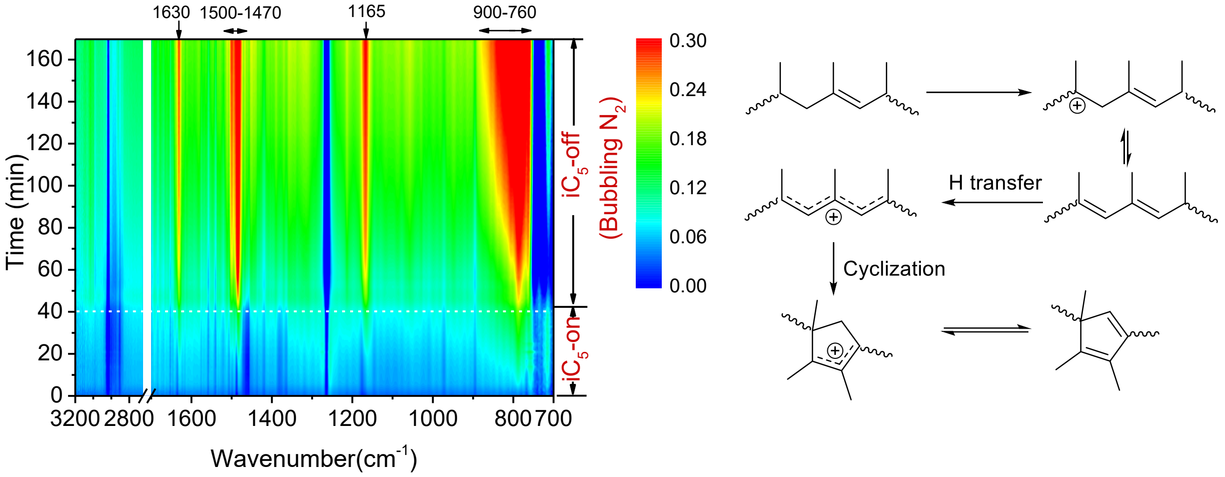

Supplementary Fig. 25 | Operando IR spectra of the iC₅ on-off experiment. Conditions were as follows: LDPE, 2 g; *i*C_5, 8 g; [C_4Py]Cl-2$AlCl_3$, 20 mmol; and temperature, 60 °C. Data were acquired with an automatic sample scan interval of 15s over the range of 4000-700 cm^{-1}.

Supplementary Note 3: Derivations of rate equations in the cracking-alkylation of polyolefin with iC_5 over $[C_4Py]Cl\text{-}xAlCl_3$

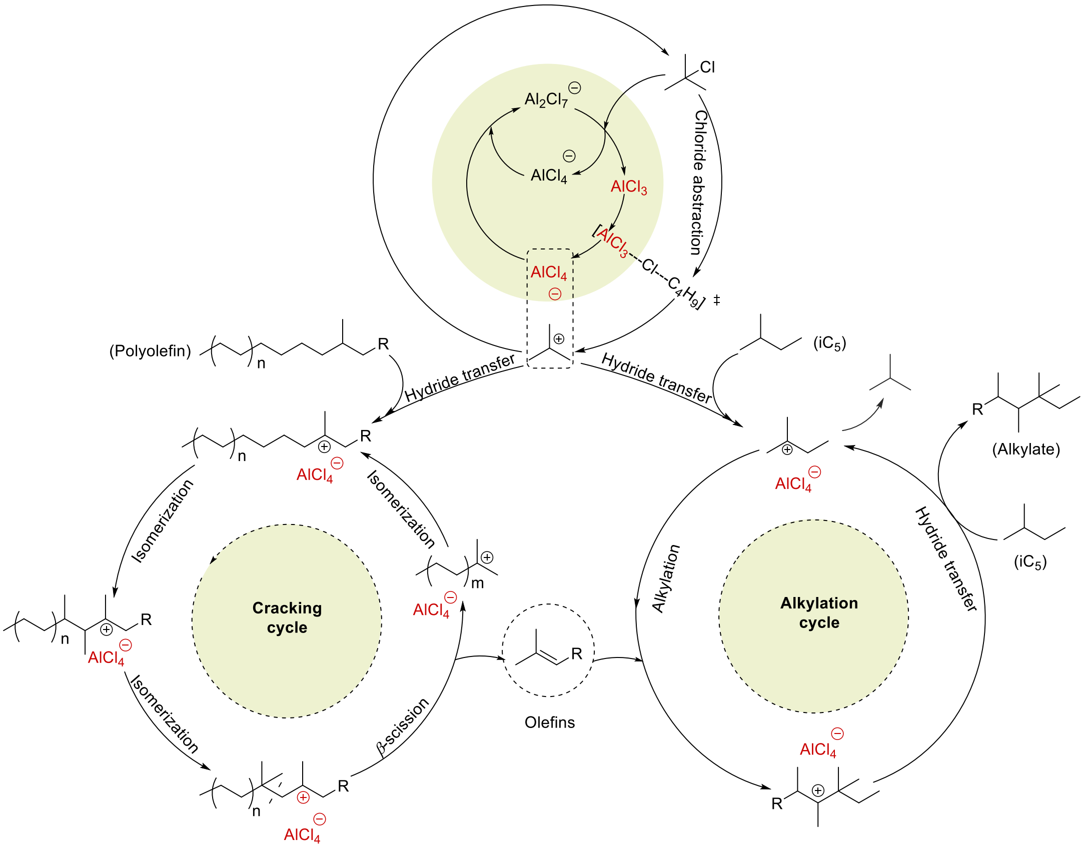

Supplementary Fig. 26 | Proposed sequence of key reaction steps in the cracking-alkylation of polyolefin with iC_5 over $[C_4Py]Cl\text{-}xAlCl_3$. All resulting carbenium ion-intermediates are not bare species but surrounded and stabilized by the counterpart $AlCl_4^-$ to form ion pairs (see details in Fig. 6 in the main text).

We propose a set of key elementary steps (shown in Fig. 6 and Supplementary Fig. 26) that involves three main stages of carbenium ions-mediated mechanism: initial formation of carbenium ions via chloride and hydride transfer, propagation of cracking and alkylation cycles of carbenium ions, and termination of carbenium ions. The overall reaction is started by small amounts of TBC reacting with $AlCl_3$ species that are in situ generated from the dissociating $Al_2Cl_7^-$, providing the initial carbenium ions to start the chain process. Then, $AlCl_4^-$-coordinated carbenium ions act as reactive ion-pair intermediates that activate the C–H bonds of hydrocarbons (either LDPE or iC_5), followed by C–H bond cleavage via hydride transfer to preferentially form tert-carbenium ions in the polymer and in the more abundant iC_5.

Next, the formed polyolefin carbenium ion-pairs undergo isomerization and cracking via *β*-scission, which yields short carbenium ions and alkenes. Simultaneously, the formed alkenes reacted with iC_5 via the alkylation step, shifting the equilibrium and catalyzing polyolefin

conversion. The long-chain fragments undergo further cracking, and alkylation cycles to the branched alkylate. Finally, the carbenium-$AlCl_4^-$ ion-pairs can be terminated either by deprotonation to an alkene followed by oligomerization (without replenishing of *i*C_5), or by recombination of hydride or chloride, i.e., the reverse of the initiation reaction (without replenishing of polyolefin) that $Al_2Cl_7^-$ regenerated again reacts with $AlCl_4^-$ to $Al_2Cl_7^-$.
Letting the overall concentrations of $AlCl_3$…adduct be $\sum[\mathrm{AlCl_3} \cdots \mathrm{adduct}]$, then we have:

$$\Delta[Al_2Cl_7{}^{\ominus}] = \Delta[AlCl_4{}^{\ominus}] = \sum[AlCl_3 \cdots adduct]$$

The proposed sequence of key reaction steps in the cracking-alkylation of polyolefin with *i*C_5 can be simplified as follows:

$$\mathrm{C_nH_{2n+2} + AlCl_3...adduct} \xrightleftharpoons{K_{a1}} \mathrm{C_nH_{2n+1}...H...AlCl_3...adduct} \quad \text{(Rxn S1)}$$

$$i\mathrm{C_5H_{12} + AlCl_3...adduct} \xrightleftharpoons{K_{a2}} i\mathrm{C_5H_{11}...H...AlCl_3...adduct} \quad \text{(Rxn S2)}$$

$$\mathrm{C_nH_{2n+1}...H...AlCl_3...adduct} \xrightleftharpoons{K_{H1}} \mathrm{C_nH_{2n+1}^{\oplus} + \{AlCl_3...adduct...H\}^{\ominus}} \quad \text{(Rxn S3)}$$

$$i\mathrm{C_5H_{11}...H...AlCl_3...adduct} \xrightleftharpoons{K_{H2}} \mathrm{iC_5H_{11}^{\oplus} + \{AlCl_3...adduct...H\}^{\ominus}} \quad \text{(Rxn S4)}$$

$$\mathrm{C_nH_{2n+1}^{\oplus} + \{AlCl_3...adduct...H\}^{\ominus}} \underset{k_{oligom}}{\overset{k_{crack}}{\rightleftharpoons}} \mathrm{C_xH_{2x}^{=} + C_{n\text{-}x}H_{2(n\text{-}x)+1}^{\oplus} + \{AlCl_3...adduct...H\}^{\ominus}} \quad \text{(Rxn S5)}$$

$$\mathrm{C_xH_{2x}^{=} + iC_5H_{11}^{\oplus} + \{AlCl_3...adduct...H\}^{\ominus}} \xrightarrow{k_{alkyl}} \mathrm{C_{x+5}H_{2x+12} + AlCl_3...adduct} \quad \text{(Rxn S6)}$$

$$\mathrm{AlCl_3...adduct} \longleftrightarrow \mathrm{C_i^{\oplus} + AlCl_4^{\ominus}} \qquad \mathrm{\{AlCl_3...adduct...H\}^{\ominus}} \longleftrightarrow \mathrm{C_i + AlCl_4^{\ominus}}$$

where active $AlCl_3$-adducts with PE and *i*C_5 (Rxn S1-S2) and the subsequent C–H bond cleavages (Rxn S3-S4) are proposed to be rapid and quasi-equilibrated, $\mathrm{K_{a1}}$, $\mathrm{K_{a2}}$, $\mathrm{K_{H1}}$ and $\mathrm{K_{H2}}$ are the reaction equilibrium constant, respectively. We have:

$$K_{a1} = \frac{[\, C_nH_{2n+1} \cdots H \cdots AlCl_3 \cdots adduct]}{[C_nH_{2n+2}][AlCl_3 \cdots adduct]} \qquad \text{(Eq. S1)}$$

$$K_{a2} = \frac{[iC_5H_{11} \cdots H \cdots AlCl_3 \cdots adduct]}{[iC_5H_{12}][AlCl_3 \cdots adduct]} \qquad \text{(Eq. S2)}$$

$$K_{H1} = \frac{[C_nH_{2n+1}{}^{\oplus}][AlCl_3 \cdots adduct \cdots H\}^{\ominus}]}{[\, C_nH_{2n+1} \cdots H \cdots AlCl_3 \cdots adduct\,]} \qquad \text{(Eq. S3)}$$

$$K_{H2} = \frac{[iC_5H_{11}{}^{\oplus}][AlCl_3 \cdots adduct \cdots H\}^{\ominus}]}{[iC_5H_{11} \cdots H \cdots AlCl_3{}^{\dagger}]} \qquad \text{(Eq. S4)}$$

We reasoned that cracking and alkylation occurred simultaneously and proceeded constantly. Because the activated hydrocarbon strands of polymers will crack at positions that are determined by the rates of hydride shift within the chain. Increasing the hydride shift rate compared to cracking will lead to longer cracked fragments and heavier products; on the contrary, the relatively higher rate of cracking will give more alkenes, facilitating the formation of acid-soluble oil via oligomerization (as the alkylation is a significantly slower reaction than the oligomerization of the alkenes). Thus, the initial reaction rate (r) can be expressed as:

$$\begin{aligned} r &= k_{crack}[C_nH_{2n+1}{}^{\oplus}][AlCl_3 \cdots adduct \cdots H\}^{\ominus}] \\ &\quad - k_{oligom}[C_{n-x}H_{2(n-x)+1}{}^{\oplus}][AlCl_3 \cdots adduct \cdots H\}^{\ominus}][C_xH_{2x}{}^{=}] \\ &= k_{alkyl}\,[iC_5H_{11}{}^{\oplus}][AlCl_3 \cdots adduct \cdots H\}^{\ominus}][C_xH_{2x}{}^{=}] \end{aligned} \quad \text{(Eq. S5)}$$

Note that the long-chain fragments (C_{n-x}) underwent further cracking and alkylation cycles. The rates of both associations of $AlCl_3$-adducts with C_{n-x} and the subsequent C–H bond cleavages were identical to that Rxn S1 and Rxn S3, then we have

$$K_{a1} = \frac{[C_{n-x}H_{2(n-x)+1} \cdots H \cdots AlCl_3 \cdots adduct]}{[C_{n-x}H_{2(n-x)+2}][AlCl_3 \cdots adduct]} \quad \text{(Eq. S6)}$$

$$K_{H1} = \frac{[C_{n-x}H_{2(n-x)+1}{}^{\oplus}][AlCl_3 \cdots adduct \cdots H\}^{\ominus}]}{[C_{n-x}H_{2(n-x)+1} \cdots H \cdots AlCl_3 \cdots adduct]} \quad \text{(Eq. S7)}$$

Combing Equations S1-S4 and S6-7, gives:

$$[C_xH_{2x}{}^{=}] = \frac{k_{crack}K_{H1}K_{a1}[C_nH_{2n+2}]}{k_{alkyl}K_{H2}K_{a2}[iC_5H_{12}] + k_{oligom}K_{H1}K_{a1}[C_{n-x}H_{2(n-x)+2}]} \quad \text{(Eq. S8)}$$

We assumed that in the kinetic regime, the concentration of C_nH_{2n+2} is identical to $C_{n-x}H_{2(n-x)+2}$, then we have

$$[C_xH_{2x}{}^{=}] = \frac{k_{crack}K_{H1}K_{a1}[C_nH_{2n+2}]}{k_{alkyl}K_{H2}K_{a2}[iC_5H_{12}] + k_{oligom}K_{H1}K_{a1}[C_nH_{2n+2}]} \quad \text{(Eq. S9)}$$

Overall, Taking Equation S7 to Equation S5, the rate equation can be expressed as:

$$\begin{aligned} r &= k_{alkyl}\,[iC_5H_{11}{}^{\oplus}][AlCl_3 \cdots adduct \cdots H\}^{\ominus}][C_xH_{2x}{}^{=}] \\ &= \frac{k_{alkyl}k_{crack}K_{H1}K_{H2}K_{a1}K_{a2}[C_nH_{2n+2}][iC_5H_{12}]}{k_{alkyl}K_{H2}K_{a2}[iC_5H_{12}] + k_{oligom}K_{H1}K_{a1}[C_nH_{2n+2}]}[AlCl_3 \cdots adduct] \\ &= \frac{k_{alkyl}k_{crack}K_{H1}K_{H2}K_{a1}K_{a2}[C_nH_{2n+2}][iC_5H_{12}]}{k_{alkyl}K_{H2}K_{a2}[iC_5H_{12}] + k_{oligom}K_{H1}K_{a1}[C_nH_{2n+2}]}[AlCl_3]_{total} \end{aligned} \quad \text{(Eq. S10)}$$

The rate normalized to the total $AlCl_3$ concentration (r_{Al}) can be expressed as:

$$\begin{aligned} r_{Al} &= \frac{r}{\sum[AlCl_3 - adduct]} \\ &= \frac{k_{alkyl}k_{crack}K_{H1}K_{H2}K_{a1}K_{a2}[C_nH_{2n+2}][iC_5H_{12}]}{k_{alkyl}K_{H2}K_{a2}[iC_5H_{12}] + k_{oligom}K_{H1}K_{a1}[C_nH_{2n+2}]} \end{aligned} \quad \text{(Eq. S11)}$$

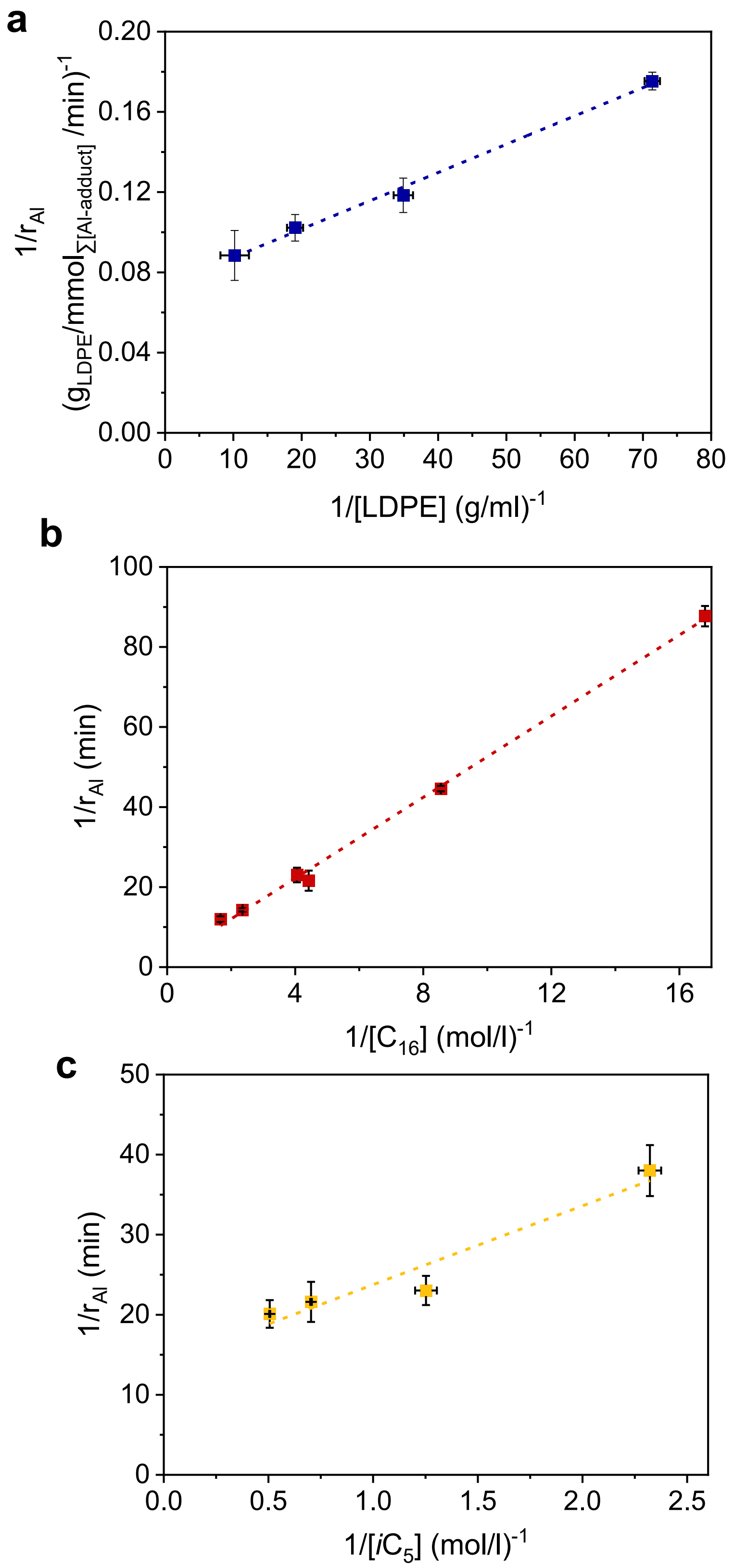

Supplementary Fig. 27 | Kinetic analysis of cracking-alkylation cycles of polyolefin with iC_5. **a-c**, Regression of the r_{Al} with concentrations of polyolefin [C_nH_{2n+2}], [C_{16}] and isopentane [iC_5H_{12}], respectively, showing excellent fits to equation (Eq. (3)) in the main text.
